# The diagnostic accuracy of lung auscultation in adult patients with acute pulmonary pathologies: a meta-analysis

**DOI:** 10.1038/s41598-020-64405-6

**Published:** 2020-04-30

**Authors:** Luca Arts, Endry Hartono Taslim Lim, Peter Marinus van de Ven, Leo Heunks, Pieter R. Tuinman

**Affiliations:** 1 Amsterdam UMC, location Vrije Universiteit Amsterdam, Department of Intensive Care Medicine, Amsterdam, The Netherlands; 2 Research Vrije Universiteit Intensive Care (REVIVE) and 4Amsterdam Cardiovascular Sciences, De Boelelaan 1117, 1081 HV, Amsterdam, The Netherlands; 3 Amsterdam UMC, Vrije Universiteit Amsterdam, Department of Biostatistics and Epidemiology, De Boelelaan 1117, 1081 HV Amsterdam, The Netherlands; 4 Amsterdam Cardiovascular Sciences, De Boelelaan 1117, 1081 HV, Amsterdam, The Netherlands

**Keywords:** Respiratory tract diseases, Respiratory signs and symptoms

## Abstract

The stethoscope is used as first line diagnostic tool in assessment of patients with pulmonary symptoms. However, there is much debate about the diagnostic accuracy of this instrument. This meta-analysis aims to evaluate the diagnostic accuracy of lung auscultation for the most common respiratory pathologies. Studies concerning adult patients with respiratory symptoms are included. Main outcomes are pooled estimates of sensitivity and specificity with 95% confidence intervals, likelihood ratios (LRs), area under the curve (AUC) of lung auscultation for different pulmonary pathologies and breath sounds. A meta-regression analysis is performed to reduce observed heterogeneity. For 34 studies the overall pooled sensitivity for lung auscultation is 37% and specificity 89%. LRs and AUC of auscultation for congestive heart failure, pneumonia and obstructive lung diseases are low, LR− and specificity are acceptable. Abnormal breath sounds are highly specific for (hemato)pneumothorax in patients with trauma. Results are limited by significant heterogeneity. Lung auscultation has a low sensitivity in different clinical settings and patient populations, thereby hampering its clinical utility. When better diagnostic modalities are available, they should replace lung auscultation. Only in resource limited settings, with a high prevalence of disease and in experienced hands, lung auscultation has still a role.

## Introduction

In 1816 Dr. Laënnec invented the most common symbol of medicine: the stethoscope^1^. The use of the stethoscope is considered an essential skill in the medical profession and is often chosen for its’ ease of use, as well as for its’ appearance and reputation^2^. Auscultation of the respiratory system is non-invasive, safe, inexpensive and easy-to-perform. History taking and a detailed physical examination, including auscultation, are considered essential parts of clinical examination. However, detailed auscultation alone can take up to 10 minutes^3^. Nowadays, physicians might not be in the position to spend that amount of time to evaluate chest sounds, potentially leading to an inefficient and superficial examination, giving a delay in further diagnostic work-up and treatment^3,4^.

To date, it is still ambiguous how this diagnostic tool contributes to the diagnostic work-up for various pulmonary entities. Despite the fact that the diagnostic accuracy of lung auscultation is widely debated, the stethoscope is still a first line diagnostic tool and used for clinical or therapeutic decision-making.

The question arises if the use of the stethoscope still attributes to further diagnostic work-up or if using the stethoscope is just a waste of time. So, is the stethoscope 200 years after its invention ready to be relegated to a museum shelf or does the stethoscope still provide vital clues to aid in the diagnosis^5,6^? The objective of this meta-analysis is to evaluate the diagnostic accuracy of lung auscultation in various clinical settings for the four most common acute respiratory pathologies: congestive heart failure, (hemato)pneumothorax, pneumonia, and obstructive lung diseases.

## Methods

### Search strategy and selection criteria

This is a systematic review and meta-analysis following PRISMA (Preferred Reporting Items for Systematic Reviews and Meta-Analyses) guidelines, to improve the quality of the meta-analysis^7^. The protocol was registered at ‘PROSPERO International prospective register of systematic reviews’ (http://www.crd.york.ac.uk/PROSPERO), registration number: CRD42016035312).

The following inclusion criteria were used:

- Study designs: case-control studies, cross-sectional studies, prospective or retrospective observational studies and randomized controlled trials.
- Time frame: all medical literature published till full search conducted on 19 January 2017.
- Participants: adult patients admitted to all clinical departments of primary or secondary care institution.
- Index test: lung auscultation, or lung auscultation as part of the physical examination.
- Comparator: all studies comparing or evaluating lung auscultation, or lung auscultation as part of the physical examination, with a reference standard mentioned below.
- Target condition: cardiopulmonary edema (refered to congestive heart failure in this meta-analysis), (hemato)pneumothorax, pneumonia, and obstructive lung diseases.
- Outcome measures: all data concerning diagnostic accuracy (sensitivity, specificity, positive and negative likelihood ratios (LRs), area under the curve (AUC) and heterogeneity). Rough data must be mentioned or retrievable.
- Reference standard: chest radiography (CXR), thoracic computed tomography (CT), Doppler echocardiography, spirometry (FEV1/FVC ratio) or final diagnosis by an expert panel, for various medical conditions.
- Language: manuscripts published in all languages.

A medical literature search specialist of the Free University medical library (J.C.F.K.) was consulted to define a robust search strategy. PubMed® Resource Guide search engine was used to access MEDLINE® database. The following terms were used (including all synonyms and closely related words) as index terms or free-text words: ‘stethoscopes’ or ‘auscultation’ or ‘respiratory system’ and ‘sensitivity’ or ‘specificity’. Supplementary Appendix A shows the complete PubMed® (MEDLINE®) search. An EMBASE® search was defined, however due to the large number of duplicates with PubMed® and disproportionate number of articles, only the extensive PubMed® search was analysed. If necessary authors were contacted for further information.

Abstracts and titles of all articles were analysed by two independent investigators (L.A. and E.H.T.L.). First all abstracts were screened using the in- and exclusion criteria described above. This step was followed by reading the remaining full text articles out of which relevant articles were selected. From a significant number of full text articles, rough data were not retrievable and these articles were excluded. The reference lists of included articles were scanned during the screening process: backward and forward citations were reviewed. Any disagreements were resolved during consensus meetings with a third reviewer (P.R.T.).

Covidence and EndNote X7® Software were used to manage the references. When described, the different breath sounds detected by the index test were also recorded with their sensitivity and specificity. To standardize nomenclature, we followed published guidelines for the definition of the different breath sounds^8,9^.

## Data Analysis

QUADAS-2 (Quality Assessment of Diagnostic Accuracy Studies) was used to assess risk of bias and applicability concerns (www.quadas.org)^10^. Supplementary Appendix B shows the form used for the QUADAS-2 assessment. Quality assessment was done by two reviewers (L.A. and E.H.T.L.) Any disagreements were resolved during consensus meetings with a third reviewer (P.R.T.).

A statistician (P.M.v.d.V.) performed statistical analysis. We selected four patient groups with the most common diagnoses in pulmonary pathology to reduce the heterogeneity encountered during the conduct of this study. Groups of pulmonary pathology included were: congestive heart failure (CHF), (hemato)pneumothorax (HPT), pneumonia, and obstructive lung diseases (OLD). Number of true positives, false positives, true negatives and false negatives were obtained from the articles and used for further analysis. As several studies considered different index tests for the same outcome in the same sample of patients, a multilevel approach accounting using the xtemelogit procedure in Stata 12® (StataCorp LLC, College Station, TX) was used to obtain pooled estimates for sensitivity and specificity and their 95% confidence interval (CI)^11^. The MIDAS command in Stata was used for forest plots and pooled estimates for LR+, LR−, diagnostic odds ratio (DOR) and Area Under The ROC curve (AUC). Deeks’ Funnel Plot asymmetry test was used to test for publication bias.

A meta-regression was performed separately for sensitivity and specificity. Predictors considered were diagnosis-group, index test used, type of department, percentage male and average age of the study sample. Univariate analyses were performed first, followed by a multivariate analyses in which all five predictors were included. Supplementary Appendix C shows extended information about the performed data analysis.

## Results

### Study selection and characteristics

**Figure 1:**
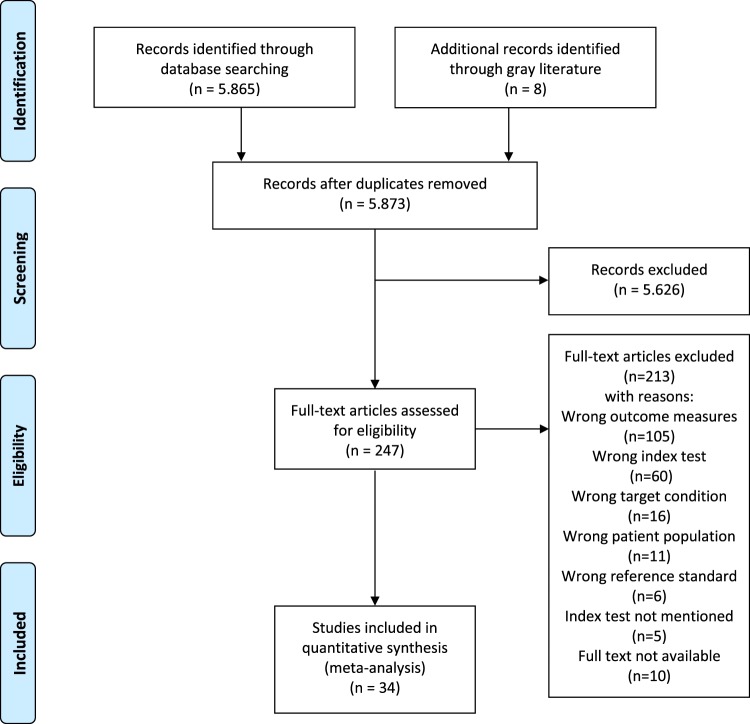
Flow chart of selection process.

After extracting the duplicates from the extended search for PubMed® (MEDLINE®), a remaining 5.873 articles were critically analysed, of which 34 were included. A large number of articles were excluded after screening the abstract, based on in- and exclusion criteria of this meta-analysis. Figure 1 shows the selection process following the PRISMA four fase flow diagram (also see supplementary Table 1 for the PRISMA checklist). Table 1 summarizes characteristics of the 34 included studies. A total of 14.814 patients were included in this analysis. Auscultation was performed by different type of investigators, with or without teaching interventions.

**Table 1:** Characteristics of included studies.

Author/Year	Diagnosis	Study design	Department/Period	Patients with… (n)	Investigator (n)
Dao *et al*.^12^	CHF	Pros.	ED, Jun-Oct 1999	Dyspnea (n = 250)	ED physician (n = ?)
Januzzi *et al*.^13^	CHF	Pros.	ED, 4 month-period	Dyspnea (n = 599)	Cardiologist (n = ?)
Knudsen *et al*.^14^	CHF	Pros.	ED (n = 7), Jun 1999-Dec 2000	Acute dyspnea (n = 880)	Research assistant (n = ?)
Knudsen *et al*.^15^	CHF	Pros.	ED,?	Acute dyspnea (n = 155)	ED resident/ cardiology fellow (n = ?)
Logeart *et al*.^16^	CHF	Pros.	ED, Jun 1999-Jun 2001	Acute dyspnea (n = 163)	ED physician (n = ?)
Morrison *et al*.^17^	CHF	Pros.	ED, Jun 1999-Jun 2000	Acute dyspnea (n = 321)	Research assistant (n = ?)
Bokhari *et al*.^18^	HPT	Pros.	ICU, Jan 2000-Jul 2001	Blunt trauma (n = 523), penetrating trauma (n = 153)	Trauma physician (n = ?)
Chen *et al*.^20^	HPT	Retrosp.	ICU, Jan-Dec 1993	Penetrating trauma (n = 118)	Surgeon (n = ?)
Chen *et al*.^19^	HPT	Pros.	ICU, Jul 1994-Aug 1996	Blunt trauma (n = 125), penetrating trauma (n = 23)	Surgeon (n = ?)
Rodriguez *et al*.^21^	HPT	Pros.	ED (n = 2), Jan 2003-May 2004	Blunt trauma (n = 492)	ED physician (n = ?)
Wormald *et al*.^22^	HPT	Pros.	Trauma unit, 5 month-period	Chest stab wounds (n = 200)	?
Badgett *et al*.^24^	OLD	Pros.	IM,?	Self-reported diagnosis of asthma, chronic bronchitis, emphysema, COPD, history of smoking (n = 92)	IM physician (n = 4)
Badgett *et al*.^23^	OLD	Pros.	IM,?	Self-reported diagnosis of asthma, chronic bronchitis, emphysema, COPD, history of smoking (n = 92)	IM physician (n = 4)
Garcia-Pachon *et al*.^29^	OLD	Pros.	PC, Feb-Jun 2001	Self-reported diagnosis of COPD, dyspnea, bronchodilator (>6 months), smoking (>20 pack-years) (n = 172)	Pulmonologist (n = 1)/ resident (n = 5)
Holleman *et al*.^25^	OLD	Pros.	IM, 12 month-period	Elective surgery (n = 164)	IM physician/ anaesthesiologist (n = 2)
King *et al*.^30^	OLD	Pros.	PC, Apr 1987-Mar 1988	Clinical suspicion of asthma with (nearly) normal spirometry (n = 44)	Physician (n = 5)
Leuppi *et al*.^26^	OLD	Pros.	ED, Nov-Dec 2001	Chest problems (n = 233)	IM physician (n = 12)
Ma *et al*.^31^	OLD	Retrosp.	RCC, 2004–2011	Acute exacerbation of bronchiectasis (n = 156)	?
Melbye *et al*.^33^	OLD	Pros.	ED		
Oct 1988-Jun 1989	Respiratory tract infection (n = 398)	Physician (n = 40)			
Pratter *et al*.^32^	OLD	Pros.	PC, 18 month-period	History of wheeze (n = 34), healthy controls (n = 7)	Pulmonologist (n = 2)
Oshaug *et al*.^27^	OLD	Cross-sectional	GP (n = 7), Apr 2009-Mar 2010	Registered diagnosis of asthma (n = 210), COPD (n = 74) or both (n = 91)	GP (n = 20)
Straus *et al*.^28^	OLD	Pros.	Healthcare center (n = 7), Apr 2009-Mar 2010	Known COPD (n = 66), suspected COPD (n = 43), without COPD (n = 52)	Physician (n = ʔ)
Tomita *et al*.^34^	OLD	Pros.	UHC, Jan 2008-Sep 2011	Non-specific respiratory symptoms (n = 566)	Pulmonologist (n = ?)
Diehr *et al*.^35^	PNA	Pros.	ED,?	Acute cough (n = 1819)	IM physician (n = ?)
Ebrahimzadeh *et al*.^36^	PNA	Case-			
control	ED, 12 month-period	Acute respiratory symptoms (n = 420)	Infectious disease specialist (n = 1)		
Gennis *et al*.^37^	PNA	Pros.	ED, Jul 1984-Feb 1985	Suspected pneumonia (n = 308)	ED/IM resident (n = ?)
Flanders *et al*.^38^	PNA	Pros.	ED, Jan-Apr 2002	Acute cough (n = 168)	?
Heckerling *et al*.^39^	PNA	Pros.	ED (n = 3), Jul 1987-Jun 1988	Respiratory symptoms (n = 1134)	Medical resident/ physician (n = ?)
Hopstaken *et al*.^40^	PNA	Pros.	GP (n = 15), Jan 1998-Apr 1999	Symptoms of lower respiratory tract infection (n = 246)	GP (n = 25)
Melbye *et al*.^41^	PNA	Pros.	ED, Oct 1988-Jun 1989	Symptoms of respiratory tract infection (n = 626)	GP (n = 40)
Minnaard *et al*.^42^	PNA	Pros.	Multicenter (n = 16), 2007–2010	Acute cough (n = 2840)	GP (n = 294)
Nakanishi *et al*.^43^	PNA	Pros.	IM/ED, Apr 2007-Mar 2009	Symptoms of lower respiratory tract infection (n = 406)	?
Reissig *et al*.^44^	PNA	Pros.	Multicenter (n = 14), Nov 2007-Feb 2011	Clinical suspicion of pneumonia (n = 362)	?
Song *et al*.^45^	PNA	Case-			
control	IM, Sep 2009- Feb 2010	Respiratory symptoms (n = 81)	?		

Abbreviations**:** CHF: congestive heart failure; HPT: (hemato)pneumothorax; OLD: Obstructive Lung Disease; Pneumonia: PNA; Pros.: Prospective observational; Retrosp.: Retrospective observational; ICU: Intensive Care Unit; ED: Emergency Department; GP: General Practitioner; IM: Internal Medicine; PC: Pulmonary Clinic; RCC: Respiratory and Critical Care Department; UHC: University hospital clinic; COPD: Chronic Obstructive Pulmonary Disease;?: Unknown.

### Diagnostic summary measures

**Figure 2:**
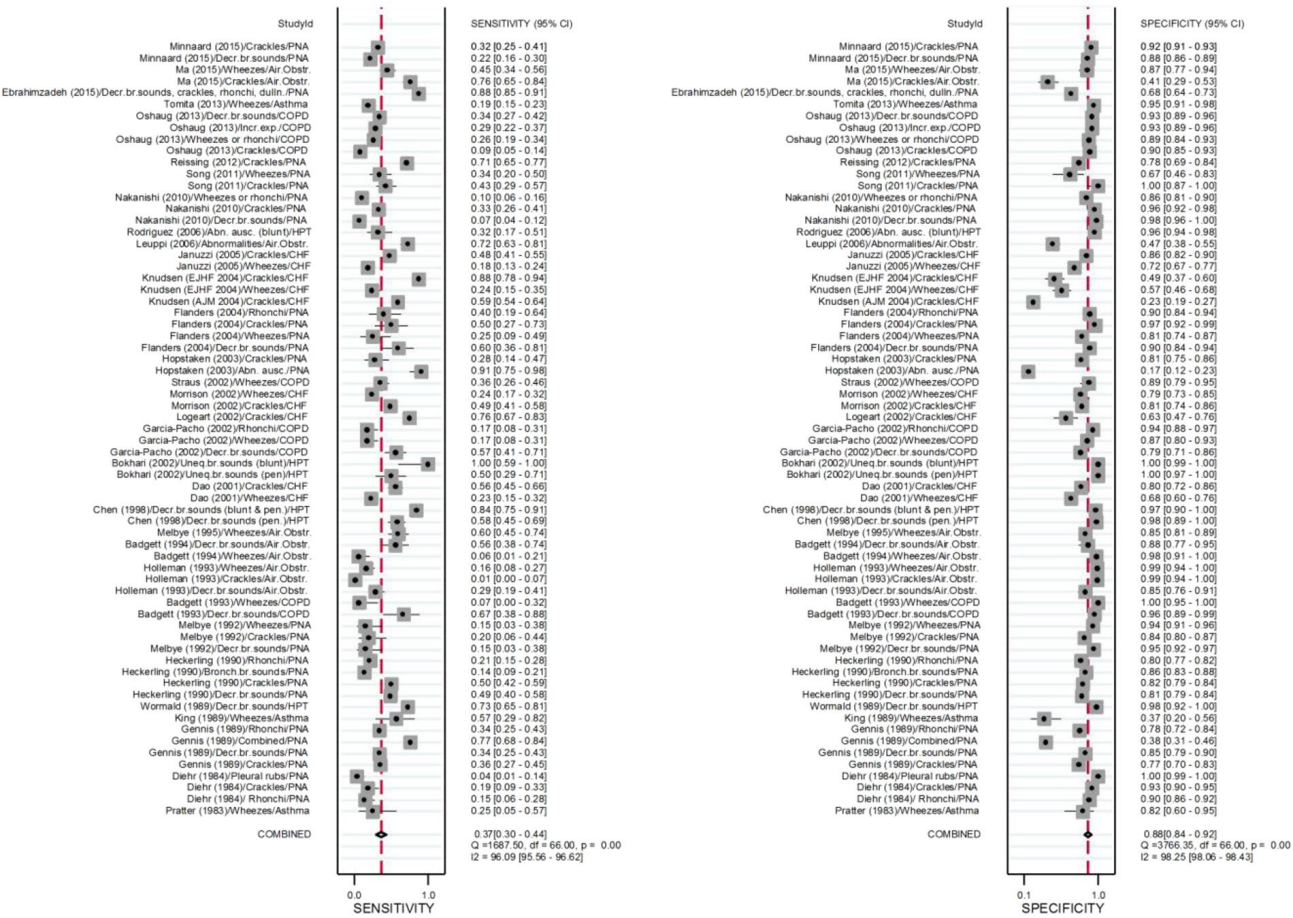
Forrest plot of sensitivity and specificity together with their 95% confidence intervals for different acute pulmonary pathology. Side note: Estimates and confidence intervals for pooled estimates may differ slightly from those in Table 2 as correlation of sensitivities (and specificities) observed for the different index-tests within the same study was ignored when making the forest-plot. Abbreviations: PNA: pneumonia; Decr. br. sounds: decreased breath sounds; Air. Obstr.: airway obstruction; dulln: dullness; COPD: chronic obstructive pulmonary disease; Abn. Ausc.: abnormal auscultation; HPT: (hemato)pneumothorax; CHF: congestive heart failure; Uneq. br. sounds: unequal breath sounds; pen.: penetrating; Air. Obstr: airway obstruction.

**Figure 3:**
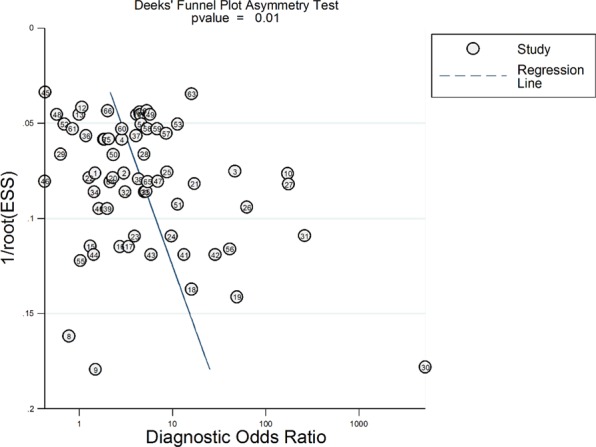
Deek’s Funnel Plot test for publication bias.

The overall pooled sensitivity for lung auscultation is 37% (95% CI: 30–47%) and specificity 89% (95% CI: 85–92%) (see Table 2 and Fig. 2). Table 3 shows the pooled estimates of sensitivity and specificity for the different types of breath sounds: abnormal, decreased or absent breath sounds, crackles, rhonchi, and wheezes. Heterogeneity was significant when considering all outcomes (P < 0.001), but also when restricted to CHF, OLD and pneumonia. Only heterogeneity of study outcomes for HPT was not significant (P = 0.38). Deeks’ Funnel Plot for all studies (Fig. 3) suggests publication bias (P = 0.01) when considering all outcomes. Publication bias was not significant, when restricting to CHF (P = 0.18), HPT (P = 0.34), OLD (P = 0.75) and pneumonia (P = 0.99). It must, however, be noted that the estimates of the bias when restricting to CHF and HPT were larger than the estimate of the bias based on all outcomes. Therefore, lack of significance for these pathology groups may be due to the small sample sizes (n = 10 and n = 6, respectively). Estimates of bias in the OLD and pneumonia subgroups were much smaller than the estimate of the bias based on all outcomes and sample sizes were larger compared to other subgroups (n = 22 and n = 29, respectively), suggesting the absence of publication bias for those pathology groups (see e-Fig. 1A-D).

**Table 2:** Diagnostic accuracy considering sensitivity, specificity, positive and negative Likelihood Ratio’s, Diagnostic Odds Ratio, and Area Under the Curve, for different pulmonary pathologies.

	Total	Sensitivity	Specificity	LR +	LR−	DOR	AUC	Heterogeneity Chi-square	I-square (95% CI)
All	34	0.37 (0.30, 0.47)	0.89 (0.85, 0.92)	3.2 (2.3, 4.2)	0.72 (0.65, 0.79)	4 (3, 6)	0.69 (0.65, 0.73)	Q = 2742, df = 2 p < 0.001	100 (100,100)
Congestive heart failure	6	0.46 (0.31, 0.62)	0.67 (0.55, 0.78)	1.4 (0.9, 2.1)	0.80 (0.59, 1.08)	2 (1,4)	0.61 (0.57, 0.65)	Q = 473.4, df=2, p < 0.001	100 (99,100)
Hematopneumothorax	5	0.70 (0.48, 0.85)	0.99 (0.97, 100)	58.2 (19.6, 173.2)	0.31 (0.16, 0.59)	190 (37, 980)	0.98 (0.97, 0.99)	Q = 0.53, df=2, p = 0.38	0 (0, 100)
Obstructive lung disease	12	0.30 (0.20, 0.42)	0.90 (0.83, 0.94)	3.0 (2.2, 4.2)	0.78 (0.69, 0.87)	4 (3,6)	0.69 (0.65, 0.73)	Q = 547.4, df=2, p < 0.001	100 (100,100)
Pneumonia	11	0.33 (0.24, 0.44)	0.87 (0.81, 0.92)	2.6 (1.9, 3.4)	0.77 (0.68, 0.87)	3 (2,5)	0.68 (0.64, 0.72)	Q = 1306.7, df=2, p < 0.001	100 (100, 100)

Abbreviations: LR: Likelihood Ratio; DOR: Diagnostic Odds Ratio; AUC: Area Under the Curve.

**Table 3:** Diagnostic accuracy for considering sensitivity, specificity, positive and negative Likelihood Ratio’s, Diagnostic Odds Ratio, and Area Under the Curve, for different breath sounds.

	Nr. studies	Sensitivity	Specificity	LR +	LR−	DOR	AUC	Heterogeneity Chi-square	I-square
**Abnormal, decreased or absent breath sounds**									
All	16	0.48 (0.34, 0.63)	0.95 (0.91, 0.97)	9.9 (4.4, 22.2)	0.54 (0.40, 0.73)	18 (6, 52)	0.86 (0.83, 0.89)	Q = 144.1 df = 2 P < 0.001	99 (98,99)
(Hemato) pneumathorax	5	0.71 (0.55, 0.83)	0.99 (0.98, 1.00)	113.5 (30.3, 425)	0.29 (0.18, 0.47)	388 (104, 1449)	0.97 (0.95, 0.98)	Q = 2.27, df = 2, p = 0.161	12 (0,100)
Obstructive lung disease	5	0.46 (0.33, 0.59)	0.89 (0.83, 0.94)	4.3 (2.4, 7.6)	0.61 (0.39, 0.78)	7 (3, 15)	0.78 (0.74, 0.81)	Q = 11.4, Df = 2, p = 0.002	82 (63, 100)
Pneumonia	6	0.26 (0.14, 0.42)	0.91 (0.84, 0.95)	2.8 (1.9, 4.1)	0.82 (0.70, 0.95)	3 (2,5)	0.73 (0.69, 0.76)	Q = 132.4, df = 2, p < 0.001	98 (98,99)
**Crackles**									
All	18	0.40 (0.27, 0.55)	0.84 (0.74, 0.91)	2.6 (1.7, 3.8)	0.71 (0.60, 0.85)	4 (2,6)	0.68 (0.64, 0.72)	Q = 1036, df = 2 p < 0.001	100 (100,100)
Congestive heart failure	6	0.64 (0.50, 0.75)	0.66 (0.45, 0.82)	1.8 (1.1, 3.1)	0.56 (0.39, 0.78)	3 (2, 7)	0.69 (0.64, 0.72)	Q = 262.7, df=2, p < 0.001	99 (99,100)
Obstructive lung disease*	3	0.14 (0.01, 0.67)	0.89 (0.41, 0.99)	1.3	0.96	1.4			
Pneumonia	9	0.35 (0.29, 0.42)	0.90 (0.84, 0.94)	3.6 (2.1, 6.1)	0.72 (0.64, 0.81)	5 (3, 9)	0.58 (0.53, 0.62)	Q = 62.967, df = 2, p < 0.001	95 (95,99)
**Rhonchi**									
All	5	0.23 (0.16, 0.31)	0.87 (0.80, 0.91)	1.7 (1.2, 2.6)	0.89 (0.81, 0.97)	2 (1,3)	0.52 (0.47, 0.56)	Q = 14.9, df =2, p < 0.001	87 (72,100)
Obstructive lung disease	Single study^‡^								
Pneumonia	4	0.25 (0.17, 0.35)	0.85 (0.79, 0.89)	1.6 (1.1, 2.5)	0.89 (0.79, 1.00)	2 (1,3)	0.57 (0.53, 0.62)	Q = 7.9, df=2, p = 0.01	75 (44, 100)
**Wheezes**									
All	17	0.24 (0.18, 0.32)	0.87 (0.87, 0.93)	1.9 (1.2, 3.1)	0.87 (0.79, 0.95)	2 (1,4)	0.48 (0.43, 0.52)	Q = 132.4, df =2, p < 0.001	98 (98,99)
Congestive heart failure	4	0.21 (0.18, 0.25)	0.70 (0.63, 0.77)	0.7 (0.5, 1.0)	1.12 (1.00, 1.25)	1 (0,1)	0.23 (0.20, 0.27)	Q = 2.6, df=2, p = 0.136	23 (0,100)
Obstructive lung disease	10	0.26 (0.15, 0.41)	0.93 (0.82, 0.97)	3.6 (1.9, 6.8)	0.79 (0.70, 0.90)	5 (2,9)	0.63 (0.58, 0.67)	Q = 110.4, df =2, p < 0.001	99 (97,99)
Pneumonia^1^	3	0.19 (0.09, 0.37)	0.85 (0.72, 0.93)	1.3	0.95	1.3			

Abbreviations: LR: Likelihood Ratio; DOR: Diagnostic Odds Ratio; AUC: Area Under the Curve. ^*^Sensitivity and specificity using xtmelogit, as Midas requires at least four studies. ^‡^Garcia-Pachon et al.^29^

### Congestive heart failure

Six prospective observational studies included patients with (acute) dyspnoea and compared auscultation with Doppler echocardiography, the Framingham criteria or by an expert panel for CHF^12–17^. Considering the results listed in Table 2, diagnostic accuracy of auscultation in patients with CHF is poor. Supplementary Figure 2 and Table 3 show that in all six studies the presence of crackles is more sensitive than the presence of wheezes for CHF.

### (Hemato)pneumothorax

Four prospective observational studies and one retrospective study included patients with blunt or penetrating chest trauma to compare auscultation with CXR for the detection of hematothorax, pneumothorax or hematopneumothorax^18–22^. Results in Table 2 show an excellent diagnostic accuracy of auscultation for HPT in trauma patients. Except for the study of Rodriques *et al*., with a low sensitivity for abnormal breath sounds in patients with HPT^21^. This is the only study that took abnormal breath sounds into account (see Supplementary Figure 3).

### Obstructive lung disease

Ten prospective observational studies, one retrospective observational study, and one cross-sectional study included patients with diagnosis of chronic obstructive lung disease (COPD) or asthma and compared auscultation with spirometry for the detection of airway obstruction^23–34^. The results listed in Table 2, show a poor diagnostic accuracy of auscultation for OLD. Table 3 shows that for the diagnosis COPD abnormal, decreased or absent breath sounds have a LR + of 4.3, with five available studies, and wheezes have a LR + of 3.6, with ten available studies (see also Supplementary Figure 4).

### Pneumonia

Nine prospective observational studies and two case-control studies included patients with acute respiratory symptoms or with an expected pneumonia and compared auscultation with CXR for the detection of pneumonia^35–45^. Table 2 shows a low diagnostic accuracy of auscultation for pneumonia in these patients. Supplementary Figure 5 demonstrates a higher sensitivity for the combination of different breath sounds, found by Ebrahimazedeh *et al*. (decreased breath sounds, crackles, rhonchi), followed by crackles as a single breath sound (see Table 3)^36^.

### Meta-regression

#### Sensitivities

In univariate analyses sensitivities were found to be associated with diagnosis-group (P < 0.001), index test used (P < 0.001), percentage male (P = 0.041) and department (P < 0.001), but not with average age of study sample (P = 0.72).

With regard to diagnosis group, sensitivities were significantly higher for HPT compared to OLD (P < 0.001) and pneumonia (P = 0.002). No other pairs of diagnosis groups were found to differ significantly in terms of sensitivity.

With regard to index text used, sensitivities were significantly higher for absent, decreased or unequal breath sounds compared to wheezes (P < 0.001) and rhonchi (P = 0.003). Sensitivities for crackles were significantly higher compared to wheezes (P < 0.001) and rhonchi (P = 0.004). No difference was found between rhonchi and wheezes (P = 1.000) and absent, decreased or unequal breath sounds and crackles (P = 1.000). With regard to departments, sensitivities were higher for Intensive Care Unit (ICU) compared to mixed patients from Emergency Department (ED) and wards (P = 0.042) or General Practice (GP), wards or ED only (P < 0.001 for all three). No differences were found in terms of sensitivity between ED, wards and GP. Sensitivity increased with 0.5% (95% CI: 0.0–0.9%) with each additional percent of males included in the study.

In a multivariate analysis including all five candidate predictors, diagnosis group no longer reached significance (P = 0.051). Index test used (P < 0.001), percentage male (P = 0.005) and department (P < 0.001) remained significantly associated with sensitivity. Sensitivities were not found to be associated with average age of study sample (P = 0.47).

#### Specificities

In univariate analyses specificities were found to be associated with diagnosis-group (P < 0.001), index test used (P = 0.013), department (P < 0.001) and average age of study sample (P = 0.001) and percentage male (P = 0.88).

With regard to diagnosis group, specificities were significantly higher for HPT compared CHF (P < 0.001) and pneumonia (P = 0.001). No other pairs of diagnosis groups were found to differ significantly in terms of specificity.

With regard to index text used, specificities were significantly higher for absent, decreased or unequal breath sounds compared to wheezes (P = 0.028). No other pairs of index tests were found to differ significantly in terms of specificity. With regard to departments, specificities were significantly higher for ICU compared ED. No other differences were found. Specificity decreased with 0.6% (95% CI: 0.3–1.0%) for each year increase in average age.

In a multivariate analysis for specificity including all five candidate predictors, only diagnosis group remained significant (P = 0.036). Specificities were not found to be associated with average age of study sample (P = 0.89), index test used (P = 0.88), percentage male (P = 0.17) and department (P = 0.22). Post-hoc tests using Bonferroni correction revealed no pairs of diagnosis groups that differed significantly in terms of their specificity.

### Risk of bias and applicability concerns

Table 4 summarizes the risk of bias and applicability assessment of included studies. Supplementary Appendix D shows complete risk of bias and applicability assessment following the QUADAS-2 guidelines. Overall, the risk of bias for most studies was considered high. Risk of bias was considered low when physicians were informed with some clinical data, assumed to be a normal clinical situation. Almost all studies matched the review question, resulting in low applicability concerns. Reasons for high risk of bias most often encountered were: a highly selected group of patients; no consecutive selection of patients, no description how selection was performed; and often patients were potentially incorrectly excluded from the analysis. Many studies did not clearly describe if the physicians performing auscultation were blinded for the reference test. The studies concerning patients with a suspected HPT and pneumonia did not use thoracic CT or final diagnosis by the treating physician, which can be considered the gold standard, but CXR as reference standard, giving a high risk of bias for the reference standard.

**Table 4:** QUADAS-2: risk of bias and applicability assessment of included studies.

Study	Risk of bias				Applicability concerns		
	Patient selection	Index test	Reference standard	Flow and timing	Patient selection	Index test	Reference standard
Dao *et al*.^12^	–	–	+	?	–	+	+
Januzzi *et al*.^13^	?	–	+	?	+	+	+
Knudsen *et al*.^14^	–	–	+	?	+	+	+
Knudsen *et al*.^15^	+	–	+	?	+	+	+
Logeart *et al*.^16^	+	–	+	?	+	+	+
Morrison *et al*.^17^	?	?	+	?	–	+	+
Bokhari *et al*.^18^	–	?	–	?	+	+	+
Chen *et al*.^20^	–	?	–	+	+	+	+
Chen *et al*.^19^	–	?	–	+	+	+	+
Rodriguez *et al*.^21^	?	–	–	–	+	–	+
Wormald *et al*.^22^	?	+	–	+	+	+	+
Badgett *et al*.^24^	–	+	?	?	+	+	+
Badgett *et al*.^23^	–	+	?	?	+	+	+
Garcia-Pachon *et al*.^29^	–	+	+	+	?	+	+
Holleman *et al*.^25^	+	+	+	+	–	+	+
King *et al*.^30^	–	+	?	+	+	+	+
Leuppi *et al*.^26^	+	+	+	+	+	+	+
Ma *et al*.^31^	–	?	?	?	+	+	+
Melbye *et al*.^33^	+	+	+	–	+	+	+
Pratter *et al*.^32^	–	+	?	?	+	+	+
Oshaug *et al*.^27^	–	?	+	?	+	+	+
Straus *et al*.^28^	–	+	+	+	+	+	+
Tomita *et al*.^34^	?	+	–	+	+	+	+
Diehr *et al*.^35^	+	?	–	?	+	+	+
Ebrahimzadeh *et al*.^36^	–	–	–	+	+	+	+
Gennis *et al*.^37^	–	–	–	?	+	+	+
Flanders *et al*.^38^	+	+	?	?	+	+	+
Heckerling *et al*.^39^	+	+	?	?	+	+	+
Hopstaken *et al*.^40^	+	+	+	+	+	+	+
Melbye *et al*.^41^	+	+	?	–	+	+	+
Minnaard *et al*.^42^	+	?	+	?	+	+	+
Nakanishi *et al*.^43^	+	+	+	–	+	+	+
Reissig *et al*.^44^	–	+	?	?	+	+	+
Song *et al*.^45^	–	–	?	?	+	+	+

+ Low;? Unclear risk; – High risk.

## Discussion

The main findings of this meta-analysis evaluating the diagnostic accuracy of lung auscultation in adult patients with acute respiratory pathology are a low sensitivity and an acceptable specificity of lung auscultation for the different pulmonary conditions studied, with an overall pooled sensitivity of 37% (95% CI: 30–47%) and specificity of 89% (95% CI: 85–92%). LRs and AUCs of auscultation for CHF, OLD and pneumonia are low. An exception is the presence of abnormal or decreased breath sounds in trauma patients, which are highly accurate for the detection of HPT. This is confirmed by multivariate analyses for specificity where diagnosis groups remained significant. Results of the meta-regression showed that the heterogeneity found could be explained by diagnosis-group, index test used, and department. We must be aware of the high risk of bias and heterogeneity reduced the quality of evidence found in this meta-analysis.

Considering the results of this meta-analysis, auscultation can be considered not clinical useful in making a diagnosis in most circumstances, based on cut-offs by Tape,T.G. (see Supplementary Appendix C), although it is hard to determine a cut-off for a minimally accepted diagnostic accuracy. Secondly, its value depends on the prevalence of the disease, clinical setting or context, and competence of the physician performing the investigation. Therefore, the different outcomes found per department can be explained by the high prevalence of disease at the ICU compared to other wards, as found in the meta-regression where sensitivities, and also specificities, were higher for patients at the ICU, compared to mixed patients from ED and wards or GP, wards or ED only. Thirdly, next to accuracy, the efficacy of auscultation also depends on how its changes clinical behaviour, e.g. how it alters clinical diagnoses and treatment decisions. For example, consider auscultation for decompensated heart failure. Crackles on auscultation have a sensitivity of 51–75% and specificity of 45–84%, carrying a LR + of 1.8 and LR− of 0.56. This limits their use in ruling decompensated heart failure in or out, because their presence of absence only marginally alters the provisional diagnosis. Although efficacy is not studied in this meta-analysis considering the overall low sensitivity, LR + and AUC, our findings suggest that lung auscultation must often be considered unfit as screening tool and for confirming a diagnosis. Especially in patients with normal auscultation and without high burden of disease, many diagnoses will go undetected and therefore additional work-up needs to be performed. In addition, it has been shown that findings from abnormal auscultation alone are insufficient to establish a diagnosis, e.g. in pneumonia and it is advised that when diagnostic certainty is required a CXR should be performed^46^. For trauma patients outside the hospital with suspected HPT an exception can be made, for which probably no further diagnostic work-up is needed, and a chest tube can be placed based on the auscultatory findings. In almost all other circumstances when auscultation is performed, still further workup is needed to conform the exact diagnosis. Fourthly, another important finding of this meta-analysis is that, although particular breath sounds are more related to a specific pathologic condition, a certain breath sound can also be present in other pulmonary diseases, lowering the diagnostic accuracy in less selected groups of patients, where the likelihood of the target condition being present is much lower. For example, decreased breath sounds which are highly specific for HPT in trauma patients, are also often found in patients with OLD or pneumonia. Fifthly, in daily practice the value of lung auscultation is further jeopardized by the experience and time of the physician performing auscultation, the subjectivity of perception and the difficulty in using standardized terminology to describe auditory findings^8,47^. As stated by Hirschtick, a “quick physical exam” is often used by the unexperienced fingers and is not much worth^47^. Lastly, a diagnostic tool can be considered obsolete when a more accurate diagnostic test is available, for example lung ultrasound which is further described below^48^.

Considering the above, we must reconsider the use of the stethoscope in patient groups with low prevalence of disease and in clinical situations where more advanced diagnostic modalities are available. Only in clinical situations in resource limited areas, with high prevalence of disease and in experienced hands the stethoscope has some clinical relevance.

### Strengths and limitations

The strengths of this meta-analysis are that it is the first on this topic, the use of a highly sensitive search strategy, a complete overview of the diagnostic accuracy of lung auscultation in a wide range of clinical settings and in predefined subgroups, and a quality assessment according to the QUADAS-2 guidelines, which is a validated and reliable instrument. When testing for publication bias, it was considered less likely. To reduce publication bias, backward citations were searched.

This meta-analysis also has weaknesses. Although, the search strategy was robust, it is still possible that not all studies were identified. Most included studies were considered to have some risk of bias. Limitations of the included studies were a wide range in number of physicians who performed auscultation, reference standards, and different clinical departments. Lastly, we changed the protocol during the conduct of the study to analyse and reduce heterogeneity.

## Further implications

We are supporters of the history and physical exam and advocate use of eyes, ears, nose and hands to study patient’s condition. However, clinicians must be progressive, embrace new modalities and let go of less reliable methods. Segall *et al*. stated in 1963: “By the year 2016, electronic systems of collecting and analysing data about the cardiovascular system may render the stethoscope obsolete.”^49^ Next to newer stethoscopes, with computerized acoustic technology which can correlate lung sounds with disease states, lung ultrasonography (LUS) has been studied extensively and seems to fulfil the role of new modality as also fantasized by Segall^49,50^. LUS, which should be seen as part of the physical examination, has many potential advantages over lung auscultation and CXR: its high accuracy, quick and easy performance and interpretation at the bedside; dynamic imaging; avoidance of radiation and contrast burden; evaluation of disease progress; and reduction of costs^51^. LUS turned out to be highly accurate for most diagnosis studied in this meta-analysis with a sensitivity and specificity of more than 90%^48,51–56^. There is also evidence showing that LUS detects respiratory problems at an early stage and impacts clinical decision making^54,57–61^. Therefore, it has been suggested before that LUS should replace lung auscultation^50,51,62^. Some important implemantations have to take place before LUS can be further implemented in today’s practice. For example, more ultrasounds devices have to be purchased and medical education has to shift its attention to ultrasonography^62^. Experts think these barriers for the implementation of LUS can relatively easily be tackled^50^, for example costs are fastly decreasing, e.g. handheld ultrasound devices are avalaible on the market for around 1500 Euro’s (1670 US dollars).

## Conclusion

This meta-analysis shows that in different patient populations with acute respiratory pathology, lung auscultation has a low sensitivity, LR + and AUC and an acceptable specificity and LR−. The results underline that auscultation only marginally alters the provisional diagnosis, although results are limited by a high risk of bias and heterogeneity of included studies. Now 200 years after the invention of the stethoscope, better diagnostic options are available such as lung ultrasound. Therefore, when better diagnostic modalities are available they should replace lung auscultation. Only in resource limited settings, with a high prevalence of disease and in experienced hands, lung auscultation has still a role.

## Supplementary information

Supplementary Table 1.

Supplementary Figures.

Supplementary Appendix A.

Supplementary Appendix B.

Supplementary Appendix C.

Supplementary Appendix D.

## Data Availability

The datasets generated during and/or analysed during the current study are available from the corresponding author on reasonable request.
