# Supplementary material for: The diagnostic accuracy of lung auscultation in adult patients with acute pulmonary pathologies: a meta-analysis
Source: Sci Rep. 2020 Apr 30;10:7347. doi: 10.1038/s41598-020-64405-6 (PMC7192898; doi:10.1038/s41598-020-64405-6)
Supplement: Supplementary file 2 — Supplementary Figures. [file 41598_2020_64405_MOESM2_ESM.pdf]

Title

The diagnostic accuracy of lung auscultation in adult patients with acute pulmonary pathologies: a meta-analysis.

Authors

Luca Arts[1,2] (MD), Endry Hartono Taslim Lim[1,2] (MD), Peter Marinus van de Ven[3] (PhD, MSc, MA), Leo Heunks[1,2,4] (PhD, MD), Pieter Roel Tuinman[1,2,4,*] (PhD, MD)

[1]Amsterdam UMC, location Vrije Universiteit Amsterdam, Department of Intensive Care Medicine, [2]Research Vrije Universiteit Intensive Care (REVIVE) and [4]Amsterdam Cardiovascular Sciences, De Boelelaan 1117, 1081 HV, Amsterdam, The Netherlands

[3]Amsterdam UMC, Vrije Universiteit Amsterdam, Department of Biostatistics and Epidemiology, De Boelelaan 1117, 1081 HV, Amsterdam, The Netherlands

Corresponding author*

Dr. P. R. Tuinman, Amsterdam UMC, Vrije Universiteit Amsterdam, Department of Intensive Care Medicine, De Boelelaan 1117, 1081 HV Amsterdam, The Netherlands

E: p.tuinman@amsterdamumc.nl, T: +31204444444

Supplementary Figure 1A – Deeks' Funnel Plot for congestive heart failure.

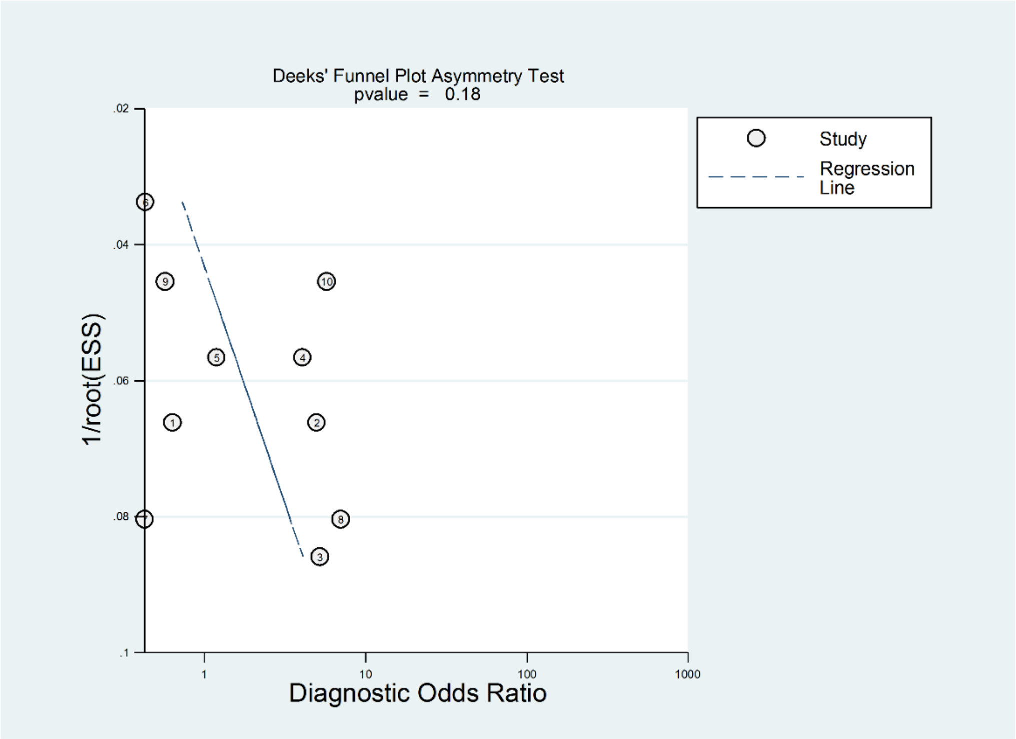

Supplementary Figure 1B – Deeks' Funnel Plot for (hemato)pneumothorax.

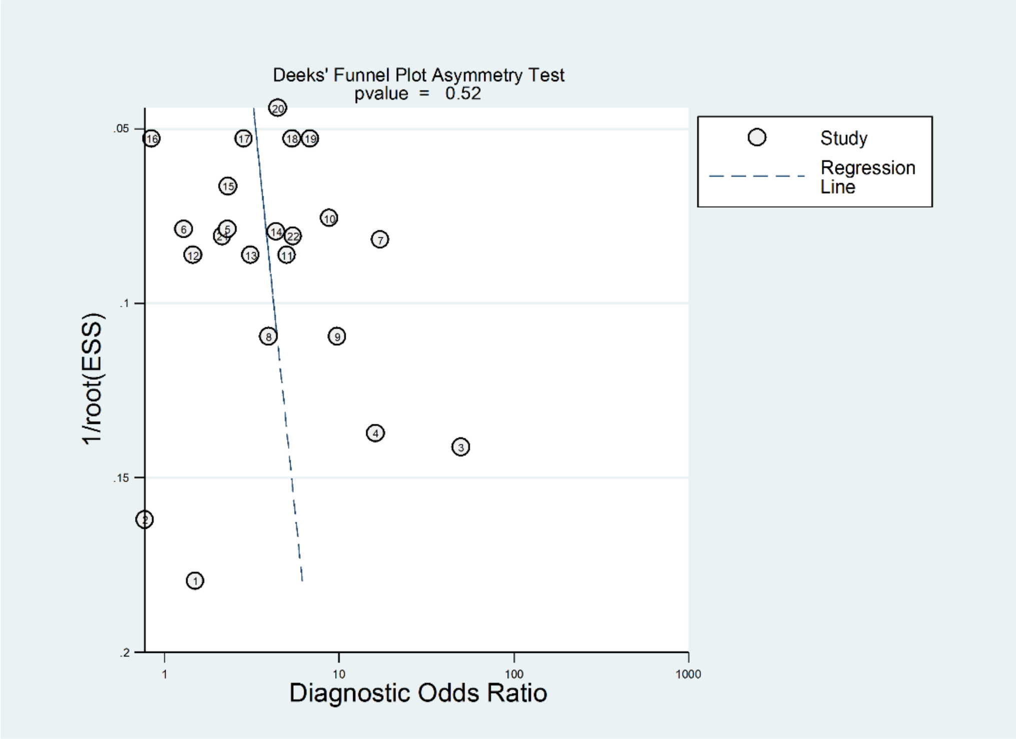

Supplementary Figure 1C – Deeks' Funnel Plot for obstructive lung disease.

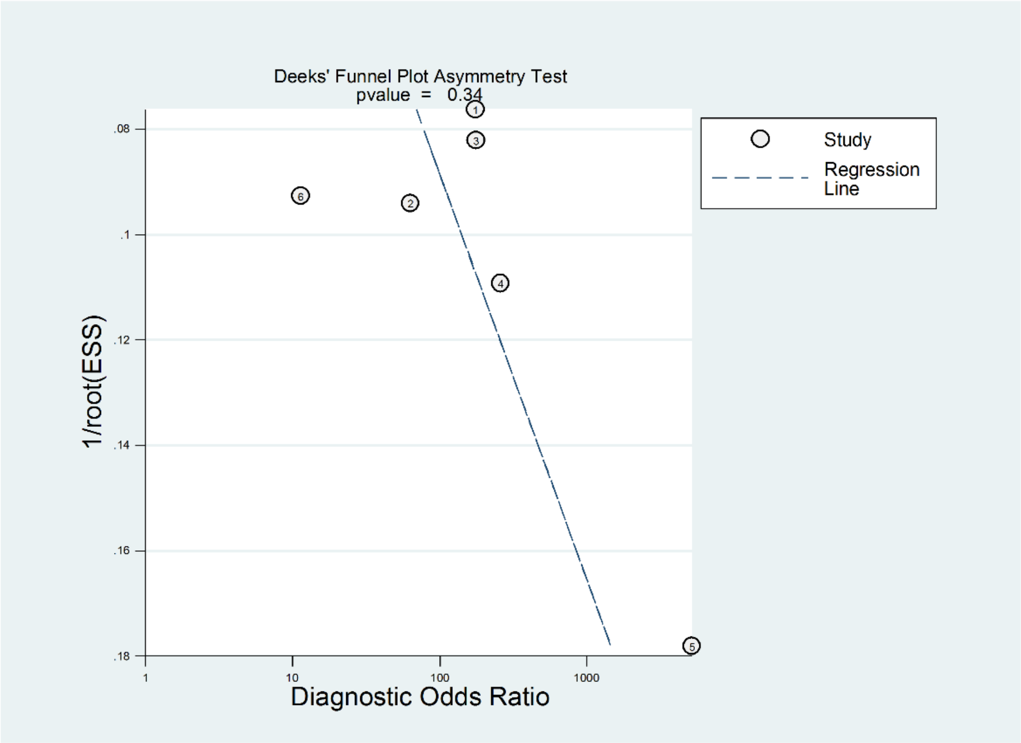

Supplementary Figure 1D – Deeks' Funnel Plot for pneumonia.

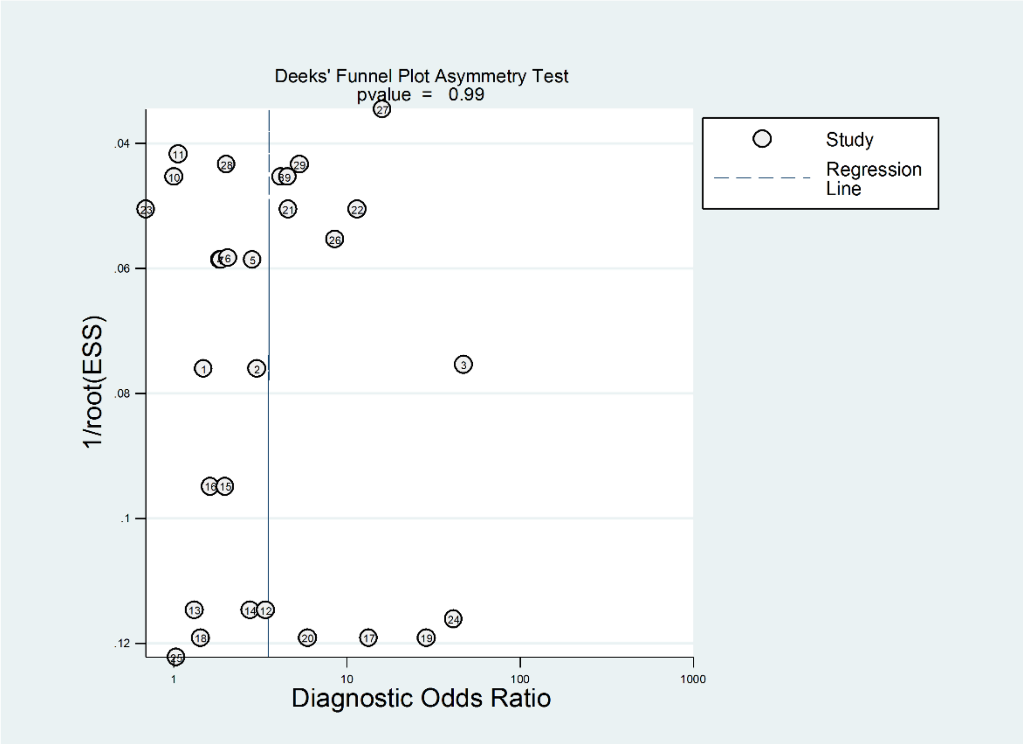

Supplementary Figure 2 – Six studies showing the range of sensitivity and specificity together with their 95% confidence intervals of different breath sounds for the detection of congestive heart failure.

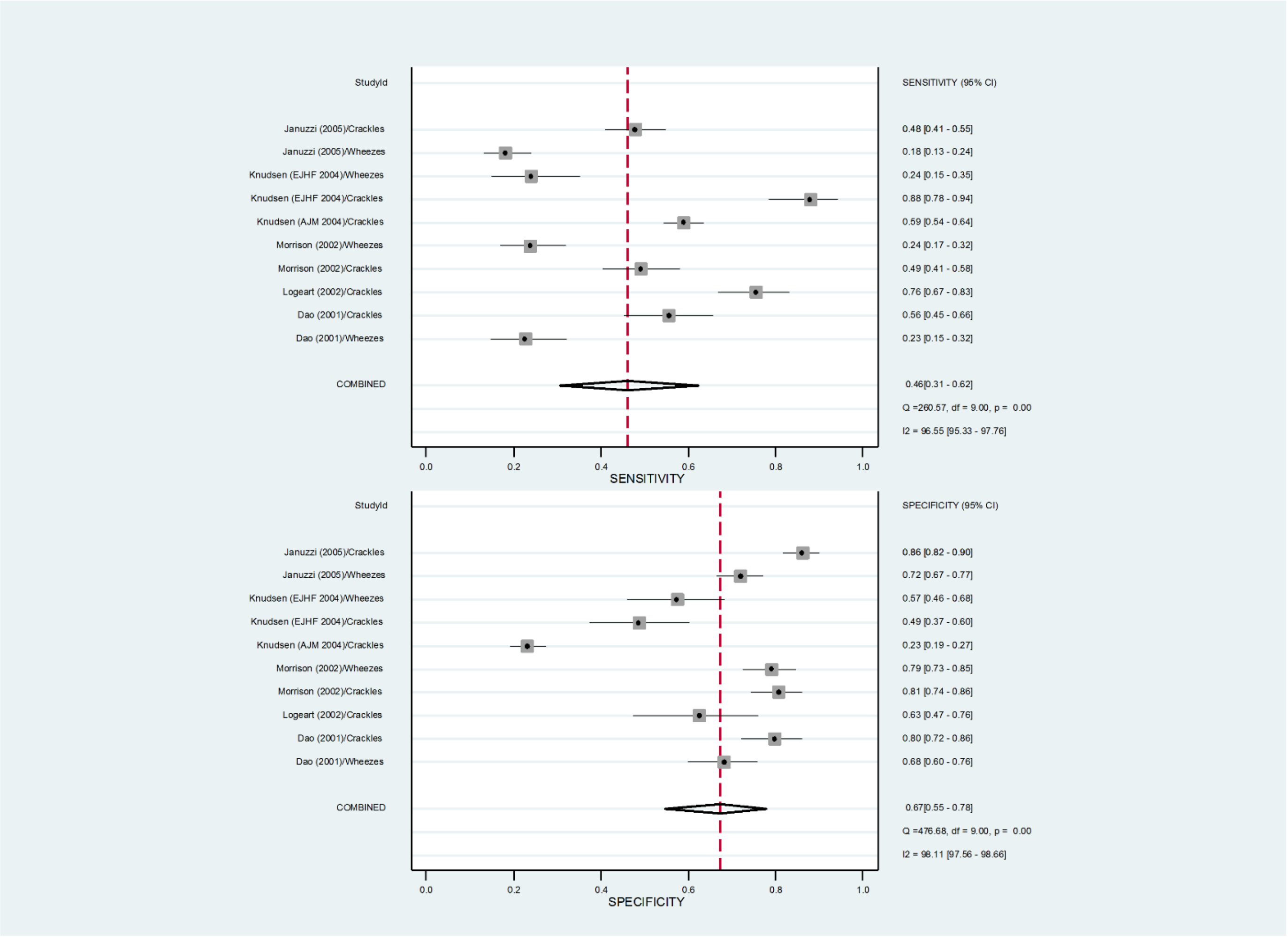

Supplementary Figure 3 – Five studies showing the range of sensitivity and specificity together with their 95% confidence intervals of different breath sounds for the detection of (hemato)pneumothorax.

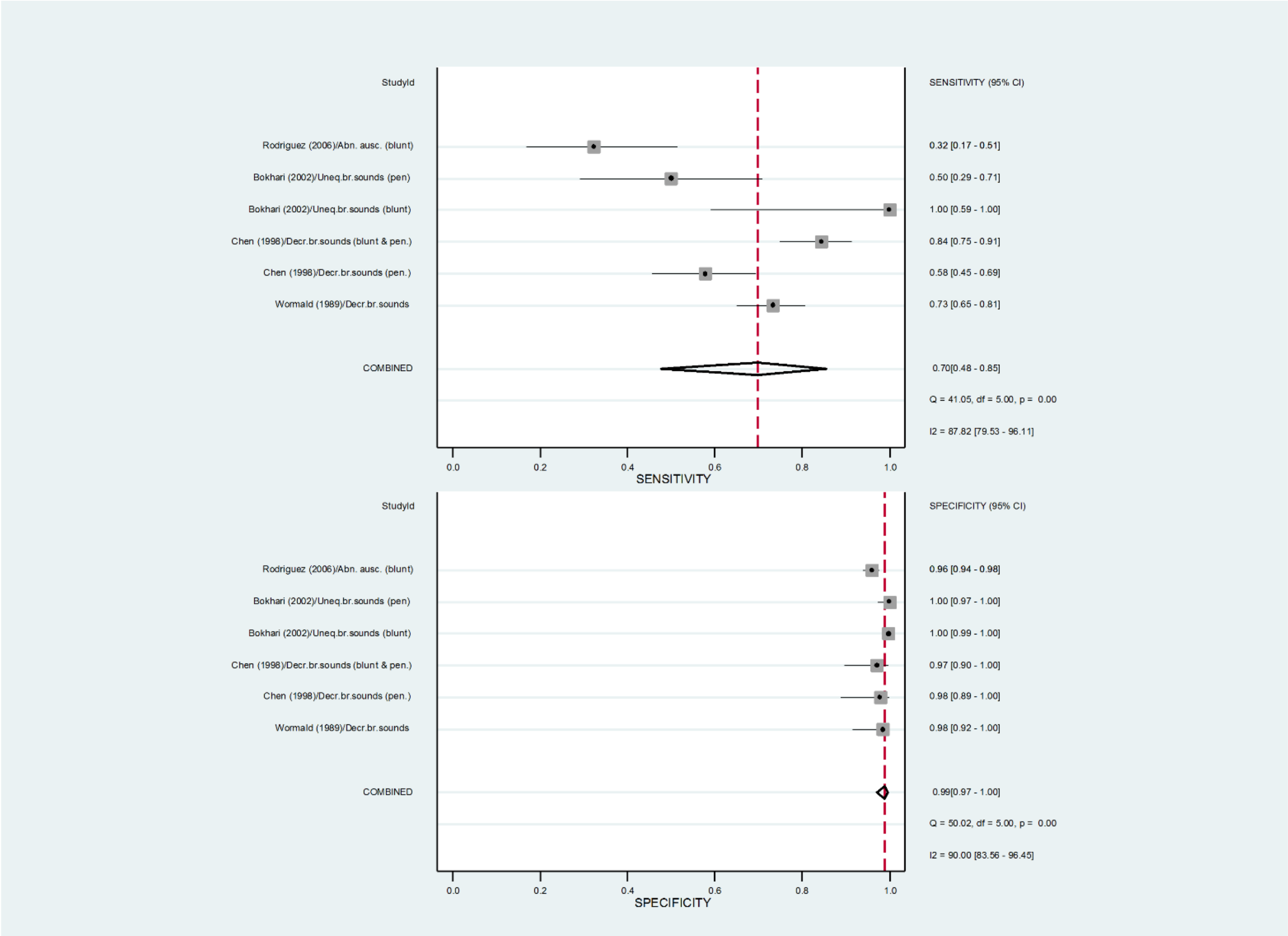

Abbreviations: Abn. ausc.: abnormal auscultation; Uneq. br. sounds: unequal breath sounds; Decr. br. sounds: decreased breath sounds; pen.: penetrating.

Supplementary Figure 4 – Eight studies showing the range of sensitivity and specificity together with their 95% confidence intervals of different breath sounds for the detection of obstructive lung disease.

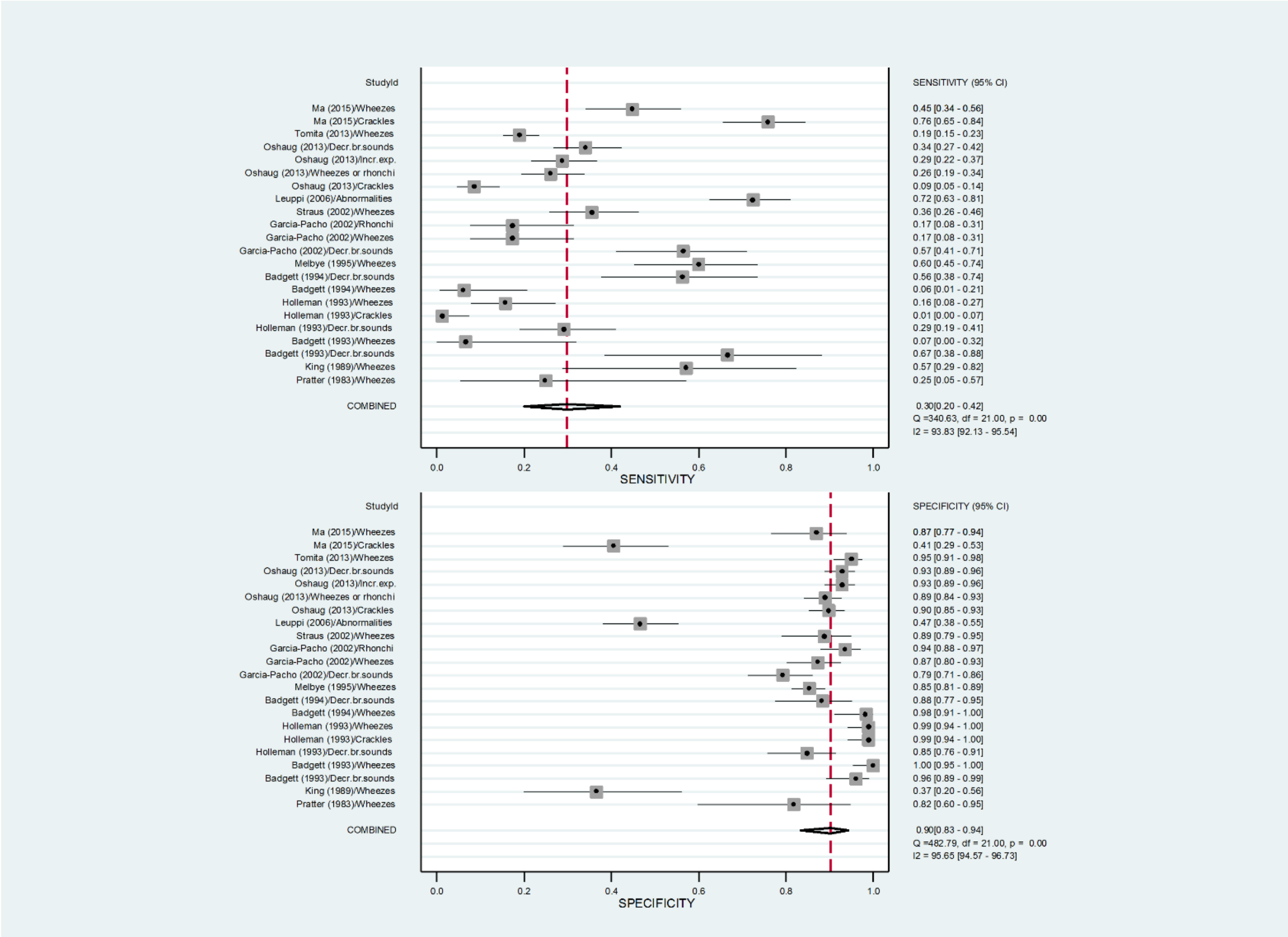

Abbreviations: COPD: chronic obstructive pulmonary disease; Incr. exp: increased expiration; Decr. br. sounds: decreased breath sounds; Air. Obstr.: airway obstruction.

Supplementary Figure 5 – Eleven studies showing the range of sensitivity and specificity together with their 95% confidence intervals of different breath sounds for the detection of pneumonia.

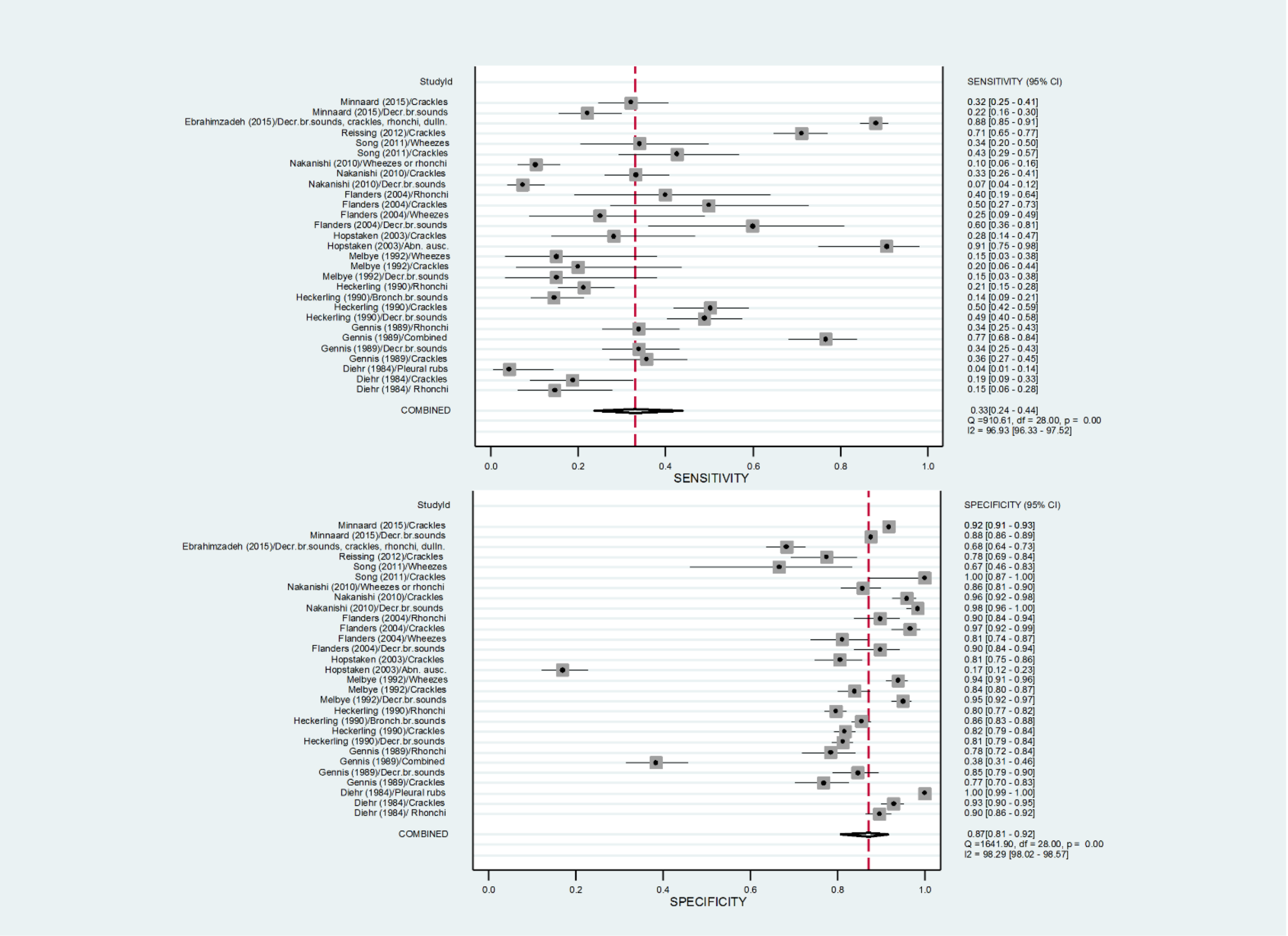

Abbreviations: Decr. br. sounds: decreased breath sounds.
