# Supplementary material for: The diagnostic accuracy of lung auscultation in adult patients with acute pulmonary pathologies: a meta-analysis
Source: Sci Rep. 2020 Apr 30;10:7347. doi: 10.1038/s41598-020-64405-6 (PMC7192898; doi:10.1038/s41598-020-64405-6)
Supplement: Supplementary file 3 — Supplementary Appendix A. [file 41598_2020_64405_MOESM3_ESM.pdf]

Title

The diagnostic accuracy of lung auscultation in adult patients with acute pulmonary pathologies: a meta-analysis.

Authors

Luca Arts[1,2] (MD), Endry Hartono Taslim Lim[1,2] (MD), Peter Marinus van de Ven[3] (PhD, MSc, MA), Leo Heunks[1,2,4] (PhD, MD), Pieter Roel Tuinman[1,2,4,*] (PhD, MD)

[1]Amsterdam UMC, location Vrije Universiteit Amsterdam, Department of Intensive Care Medicine, [2]Research Vrije Universiteit Intensive Care (REVIVE) and [4]Amsterdam Cardiovascular Sciences, De Boelelaan 1117, 1081 HV, Amsterdam, The Netherlands

[3]Amsterdam UMC, Vrije Universiteit Amsterdam, Department of Biostatistics and Epidemiology, De Boelelaan 1117, 1081 HV, Amsterdam, The Netherlands

Corresponding author*

Dr. P. R. Tuinman, Amsterdam UMC, Vrije Universiteit Amsterdam, Department of Intensive Care Medicine, De Boelelaan 1117, 1081 HV Amsterdam, The Netherlands

E: p.tuinman@amsterdamumc.nl, T: +31204444444

Supplementary Appendix A – Search strategy

PubMed® (MEDLINE®) was searched from inception (by L.A., E.H.T.L., J.C.F.K.). No filters for publication period were activated in order to retrieve all articles published since invention of the stethoscope. The Boolean operator AND, OR and NOT were used to combine search terms mentioned before to conduct a search. Duplicate articles were excluded. The following terms were used (including synonyms and closely related words) as index terms or free-text words: 'stethoscopes' or 'auscultation' or 'respiratory system' and 'sensitivity' or 'specificity'.

MEDLINE® search strategies run on 19 January 2017:

("Stethoscopes"[Mesh] OR "Auscultation"[Mesh:NoExp] OR stethoscop*[tiab] OR auscultat*[tiab] OR "Respiratory Sounds"[Mesh:NoExp] OR breath sound*[tiab] OR rale[tiab] OR rales[tiab] OR rhonchi[tiab] OR crackle*[tiab] OR respiratory sound*[tiab] OR breathing sound*[tiab] OR lung sound*[tiab] OR rhonchus[tiab] OR wheez*[tiab]) AND (Diagnosis/Narrow[filter] OR "Sensitivity and Specificity"[MeSH] OR "Diagnostic Errors"[MeSH] OR sensitive[tw] OR sensitivity[tw] OR specificity[tw] OR accurate[tw] OR accuracy[tw] OR "golden standard" OR "gold standard" OR (reference[tw] AND (test[tw] OR standard[tw])) OR "index test" OR validity[tw] OR validation[tw] OR validate*[tw] OR valid[ti] OR validation studies[pt] OR verif*[ti] OR evaluation studies[pt] OR evaluat*[ti] OR (false[tw] AND (positive[tw] OR negative[tw])) OR pretest[tw] OR pre-test[tw] OR posttest[tw] OR post-test[tw] OR predictive value OR predict*[ti] OR roc[tw] OR likelyhood[tw] OR likelihood[tw] OR value*[ti] OR reference values[mesh] OR cutoff[tw] OR cut-off[tw] OR quality control[mesh] OR "reproducibility of results"[mesh] OR repeatability[tw] OR reproducibility[tw] OR efficacy[tw] OR reliability[tw] OR comparative study[pt] OR odds[tw] OR error*[tw] OR suitability[tw] OR utility[tw]) AND ("Respiratory System"[Mesh] OR "Respiratory Tract Diseases"[Mesh] OR lung[tiab] OR lungs[tiab] OR pulmonar*[tiab] OR trachea*[tiab] OR alveol*[tiab] OR bronchi*[tiab] OR bronchus[tiab] OR respirator*[tiab] OR COPD*[tiab] OR chronic obstructive pulmonary disease*[tiab] OR COAD*[tiab] OR chronic obstructive airway disease*[tiab] OR chronic airflow obstruction*[tiab] OR pneumoni*[tiab] OR bronchopneumoni*[tiab] OR lung inflammati*[tiab] OR pleuropneumoni*[tiab] OR congestive heart failure*[tiab] OR chf*[tiab] OR pneumothora*[tiab] OR hemopneumothora*[tiab] OR haemopneumothora*[tiab]): **5.865 results (after removing duplicates)**
