# Supplementary material for: The diagnostic accuracy of lung auscultation in adult patients with acute pulmonary pathologies: a meta-analysis
Source: Sci Rep. 2020 Apr 30;10:7347. doi: 10.1038/s41598-020-64405-6 (PMC7192898; doi:10.1038/s41598-020-64405-6)
Supplement: Supplementary file 4 — Supplementary Appendix B. [file 41598_2020_64405_MOESM4_ESM.pdf]

Title

The diagnostic accuracy of lung auscultation in adult patients with acute pulmonary pathologies: a meta-analysis.

Authors

Luca Arts[1,2] (MD), Endry Hartono Taslim Lim[1,2] (MD), Peter Marinus van de Ven[3] (PhD, MSc, MA), Leo Heunks[1,2,4] (PhD, MD), Pieter Roel Tuinman[1,2,4,*] (PhD, MD)

[1]Amsterdam UMC, location Vrije Universiteit Amsterdam, Department of Intensive Care Medicine, [2]Research Vrije Universiteit Intensive Care (REVIVE) and [4]Amsterdam Cardiovascular Sciences, De Boelelaan 1117, 1081 HV, Amsterdam, The Netherlands

[3]Amsterdam UMC, Vrije Universiteit Amsterdam, Department of Biostatistics and Epidemiology, De Boelelaan 1117, 1081 HV, Amsterdam, The Netherlands

Corresponding author*

Dr. P. R. Tuinman, Amsterdam UMC, Vrije Universiteit Amsterdam, Department of Intensive Care Medicine, De Boelelaan 1117, 1081 HV Amsterdam, The Netherlands

E: p.tuinman@amsterdamumc.nl, T: +31204444444

Supplementary Appendix B – QUADAS-2: Risk of bias and applicability judgments: the four domains.

DOMAIN 1: PATIENT SELECTION

A. Risk of Bias

Describe methods of patient selection:

Was a consecutive or random sample of patients enrolled?	Yes/No/Unclear
Was a case-control design avoided?	Yes/No/Unclear
Did the study avoid inappropriate exclusions?	Yes/No/Unclear

Could the selection of patients have introduced bias? **RISK: LOW/HIGH/UNCLEAR**

B. Concerns regarding applicability

Describe included patients (prior testing, presentation, intended use of index test and setting):

Is there concern that the included patients do not match the review question? **CONCERN: LOW/HIGH/UNCLEAR**

DOMAIN 2: INDEX TEST(S)

If more than one index test was used, please complete for each test.

A. Risk of Bias

Describe the index test and how it was conducted and interpreted:

Were the index test results interpreted without knowledge of the results of the reference standard?	Yes/No/Unclear
If a threshold was used, was it pre-specified?	Yes/No/Unclear

Could the conduct or interpretation of the index test have introduced bias? **RISK: LOW /HIGH/UNCLEAR**

B. Concerns regarding applicability

Is there concern that the index test, its conduct, or interpretation differ from the review question **CONCERN: LOW/HIGH/UNCLEAR**

DOMAIN 3: REFERENCE STANDARD

A. Risk of Bias

Describe the reference standard and how it was conducted and interpreted:

Is the reference standard likely to correctly classify the target condition? Yes/No/Unclear

Were the reference standard results interpreted without knowledge of the results of the index test? Yes/No/Unclear

Could the reference standard, its conduct, or its interpretation have introduced bias? **RISK: LOW /HIGH/UNCLEAR**

B. Concerns regarding applicability

Is there concern that the target condition as defined by the reference standard does not match the review question? **CONCERN: LOW /HIGH/UNCLEAR**

DOMAIN 4: FLOW AND TIMING

A. Risk of Bias

Describe any patients who did not receive the index test(s) and/or reference standard or who were excluded from the 2x2 table (refer to flow diagram):

Describe the time interval and any interventions between index test(s) and reference standard:

Was there an appropriate interval between index test(s) and reference standard? Yes/No/Unclear

Did all patients receive a reference standard? Yes/No/Unclear

Did patients receive the same reference standard? Yes/No/Unclear

Were all patients included in the analysis? Yes/No/Unclear

Could the patient flow have introduced bias? **RISK: LOW /HIGH/UNCLEAR**
