# Supplementary material for: The diagnostic accuracy of lung auscultation in adult patients with acute pulmonary pathologies: a meta-analysis
Source: Sci Rep. 2020 Apr 30;10:7347. doi: 10.1038/s41598-020-64405-6 (PMC7192898; doi:10.1038/s41598-020-64405-6)
Supplement: Supplementary file 5 — Supplementary Appendix C. [file 41598_2020_64405_MOESM5_ESM.pdf]

Title

The diagnostic accuracy of lung auscultation in adult patients with acute pulmonary pathologies: a meta-analysis.

Authors

Luca Arts[1,2] (MD), Endry Hartono Taslim Lim[1,2] (MD), Peter Marinus van de Ven[3] (PhD, MSc, MA), Leo Heunks[1,2,4] (PhD, MD), Pieter Roel Tuinman[1,2,4,*] (PhD, MD)

[1]Amsterdam UMC, location Vrije Universiteit Amsterdam, Department of Intensive Care Medicine, [2]Research Vrije Universiteit Intensive Care (REVIVE) and [4]Amsterdam Cardiovascular Sciences, De Boelelaan 1117, 1081 HV, Amsterdam, The Netherlands

[3]Amsterdam UMC, Vrije Universiteit Amsterdam, Department of Biostatistics and Epidemiology, De Boelelaan 1117, 1081 HV, Amsterdam, The Netherlands

Corresponding author*

Dr. P. R. Tuinman, Amsterdam UMC, Vrije Universiteit Amsterdam, Department of Intensive Care Medicine, De Boelelaan 1117, 1081 HV Amsterdam, The Netherlands

E: p.tuinman@amsterdamumc.nl, T: +31204444444

Supplementary Appendix C – Data analysis

A rough guide for classifying the diagnostic accuracy by using the Area Under the Curve (AUC) of a diagnostic test is the traditional academic point system by Tape, T.G.: excellent (0.90-1.0), good (0.80-0.90), fair (0.70-0.80), poor (0.60-0.70) or fail (0.50-0.60).(11) Diagnostic summary measures were obtained for the whole set of outcomes and index tests, as well as for the separate groups based on the pulmonary pathology. In addition, summary measures were obtained for different categories of breath sounds.

Generalized estimating equations were used to account for possible correlation between sensitivities (respectively specificities) for different index tests obtained in a single study. An identity link was used together with an exchangeable correlation structure. In case a categorical variable was found to be significantly associated with sensitivity (or specificity) pairwise comparisons were made between categories using a Bonferroni correction to take into account multiple testing.
