# Supplementary material for: The diagnostic accuracy of lung auscultation in adult patients with acute pulmonary pathologies: a meta-analysis
Source: Sci Rep. 2020 Apr 30;10:7347. doi: 10.1038/s41598-020-64405-6 (PMC7192898; doi:10.1038/s41598-020-64405-6)
Supplement: Supplementary file 6 — Supplementary Appendix D. [file 41598_2020_64405_MOESM6_ESM.pdf]

Title

The diagnostic accuracy of lung auscultation in adult patients with acute pulmonary pathologies: a meta-analysis.

Authors

Luca Arts[1,2] (MD), Endry Hartono Taslim Lim[1,2] (MD), Peter Marinus van de Ven[3] (PhD, MSc, MA), Leo Heunks[1,2,4] (PhD, MD), Pieter Roel Tuinman[1,2,4,*] (PhD, MD)

[1]Amsterdam UMC, location Vrije Universiteit Amsterdam, Department of Intensive Care Medicine, [2]Research Vrije Universiteit Intensive Care (REVIVE) and [4]Amsterdam Cardiovascular Sciences, De Boelelaan 1117, 1081 HV, Amsterdam, The Netherlands

[3]Amsterdam UMC, Vrije Universiteit Amsterdam, Department of Biostatistics and Epidemiology, De Boelelaan 1117, 1081 HV, Amsterdam, The Netherlands

Corresponding author*

Dr. P. R. Tuinman, Amsterdam UMC, Vrije Universiteit Amsterdam, Department of Intensive Care Medicine, De Boelelaan 1117, 1081 HV Amsterdam, The Netherlands

E: p.tuinman@amsterdamumc.nl, T: +31204444444

Supplementary Appendix D – Risk of bias assessment

Dao et al.[17]

Selection: The risk of selection bias is high, because patient recruitment has been done by convenience sampling. Applicability concerns are high, because 94% of included patients were male.

Index test: The risk of bias is high because, it is unclear if the emergency physicians who were performing physical examination were blinded for CXR results. Moreover, no criteria for auscultation were mentioned. Applicability concerns were low.

Reference test: Two cardiologists reviewed all medical records pertaining to the patient, made independent initial assessments of the probability of each patient having CHF and were blinded to emergency physician diagnosis. The risk of bias is low, because the diagnosis of CHF was based on independent confirmation of two cardiologists and was based on generally accepted Framingham criteria. Applicability concerns are low.

Flow and timing: The risk of bias is unclear, because it is unclear when auscultation and CXR were performed.

Januzzi et al.[18]

Selection: The risk of selection bias is unlcear, because it is unclear if patient recruitment was consecutive. Exclusions were reasonable. Applicability concerns are low, because included patients match the review question.

Index test: The risk of bias is high, because is is unclear if the emergency physician who was performing physical examination was blinded for CXR results. Moreover, no criteria for auscultation were mentioned. Applicability concerns were low.

Reference test: The final diagnosis was determined for each patient by study cardiologists who were provided with all hospital records. The risk of bias is low, because the diagnosis of CHF was based on independent confirmation of two cardiologists and was based on generally accepted Framingham criteria. Applicability concerns are low.

Flow and timing: The risk of bias is unclear, because it is unclear when auscultation and CXR were performed.

Knudsen et al.[19]

Selection: The risk of selection bias is high, because patients with incomplete data were excluded. Applicability concerns are low, because included patients match the review question.

Index test: The risk of bias is high, because is is unclear if the trained research personnell who was performing physical examination were blinded for CXR results. Moreover, no criteria for auscultation were mentioned. Applicability concerns are low.

Reference test: The risk of bias is low, because the diagnosis of CHF was based on independent confirmation of two cardiologists and was based on generally accepted Framingham criteria. Applicability concerns are low.

Flow and timing: The risk of bias is unclear, because it is unclear when auscultation and CXR were performed.

Knudsen et al.[20]

Selection: The risk of selection bias is low, because patient recruitment was consecutive and exclusions were reasonable. Applicability concerns are low, because included patients match the review question.

Index test: The risk of bias is high, because all patients were examined by the medical resident or cardiology fellow on duty according to common clinical practice, but is was unlcear if they were blinded for CXR results. Moreover, no criteria for auscultation were mentioned. Applicability concerns are low.

Reference test: The final diagnosis was determined for each patient by two cardiologist. If there was disagreement about the final diagnosis, further diagnostic workup was obtained. The risk of bias is low, because the diagnosis of CHF was based on independent confirmation of two cardiologists and was based on generally accepted Framingham criteria. Applicability concerns are low.

Flow and timing: The risk of bias is unclear, because it is unclear when auscultation and CXR were performed.

Logeart et al.[21]

Selection: The risk of selection bias is low, because patient recruitment was consecutive and exclusions were reasonable. Applicability concerns are low, because included patients match the review question.

Index test: The risk of bias was high, because it is unclear if the senior physician who was performing physical examination was blinded for CXR results. Moreover, no criteria for auscultation are mentioned. Applicability concerns are low.

Reference test: The final diagnosis was determined for each patient by two cardiologists and one pneumologist, who were blinded to the results of BNP assay and Doppler echocardiography obtained on admission. The risk of bias is low, because the diagnosis of CHF was based on independent confirmation of two cardiologists and was based on generally accepted Framingham criteria. Applicability concerns are low.

Flow and timing: The risk of bias is unclear, because it is unclear when auscultation and CXR were performed.

Morrison et al.[22]

Selection: The risk of selection bias is unclear, because it is unknown if patient recruitment was consecutive. Applicability concerns are high, because 95% of included patients were male.

Index test: The risk of bias is unclear, because there was one research assistant who collected data from physical examination and it was unclear if this person was blinded for results of reference tests. Moreover, no criteria for auscultation are mentioned. Applicability concerns are low.

Reference test: The diagnosis of CHF was based on independent confirmation of two cardiologists and was based on generally accepted Framingham criteria, so risk of bias is low. Applicability concerns are low.

Flow and timing: The risk of bias is unclear, because it is unclear when auscultation and CXR were performed.

Bokhari et al.[23]

Selection: The risk of selection bias is high, because patients were not consecutive. Inclusion criteria are reasonable. Applicability concerns are low, because included patients match the review question.

Index test: The risk of bias is unclear, because it is not described if the trauma team performing auscultation was blinded to CXR results and if auscultation was performed before or after making the CXR. Moreover, if there was disagreement in the examination, the evaluation of the most senior person present was used, these changes in evaluation were not recorded. Applicability concerns are low.

Reference test: The risk of bias is high, because the trauma team who performed auscultation also evaluated the CXR. Small HPTs could have been missed, because no thoracic CT has been made. Applicability concerns are low.

Flow and timing: The risk of bias is unclear, because it is unclear when auscultation and CXR were performed.

Chen et al.[25]

Selection: The risk of selection bias is high, because patient recruitment was retrospective, patients on mechanical ventilation and patients with an incomplete record were excluded. Applicability concerns are low, because included patients match the review question.

Index test: The risk of bias is unclear, as it was not stated whether the surgeons performing auscultation were blinded to radiographic information. A treshhold for auscultatory findings is described in the introduction, but from the results section it is unclear which treshholds were used. The number of performing physicians was unclear. Applicability concerns are low.

Reference test: The risk of bias is high, because authors did not mention who evaluated the CXR and if this investigator was blinded for results of the index test. Small HPTs could have been missed, because no thoracic CT has been made. Applicability concerns are low.

Flow and timing: The risk of bias is low, because auscultation was performed immediately in the trauma resuscitation area before making the CXR.

Chen et al.[24]

Selection: The risk of selection bias is high, because patients on mechanical ventilation were excluded and it is not reported if consecutive patients were enrolled. Applicability concerns are low, because included patients match the analysis question.

Index test: The risk of bias is unclear, as it is not stated whether the surgeons performing auscultation were blinded to radiographic information. A treshhold for auscultatory findings is described in the introduction, but from the results section it is unclear which tressholds were used. The number of performing physicians is unclear. Applicability concerns are low.

Reference test: The risk of bias is high, because authors did not mention who evaluated the CXR and if this investigator was blinded for results of the index test. Small HPTs could have been missed, because no thoracic CT has been made. Applicability concerns are low.

Flow and timing: The of bias is low, because auscultation was performed immediately in the trauma resuscitation area before making the CXR.

Rodriguez et al.[26]

Selection: The risk of bias is uclear, because it is unclear if a consecutive or random sample of patients is used. Applicability concerns are low, because included patients match the review question.

Index test: The emergency medicine providers performing auscultation were blinded to radiographic information. However, risk of bias is high, because no criteria for 'abnormal chest auscultation' were determined. Moreover, the prevalence the investigators used to determine the accuracy of auscultation also contained other traumatic injuries (e.g. fractures) and not only the injuries that can be found by auscultation. Applicability concerns are high.

Reference test: An independent radiologist analysed the CXR and was blinded to study implementation. Risk of bias is high, because a thoracic CT has only been made when patients were suspected for having aortic injuries, so small HPTs could have been missed in the other cases. Applicability concerns are low.

Flow and timing: Auscultation was performed when CXR was orderd or performed. The risk of bias is high, because not all patients received the same reference standard, a few patients received an CT after CXR.

Wormald et al.[27]

Selection: The risk of bias is unclear, because it is not stated whether a consecutive sample of patients was enrolled. Applicability concerns are low, because included patients match the review question.

Index test: The risk of bias is low, because pulmonary auscultation was performed before the CXR was made. Applicability concerns are low.

Reference test: The risk of bias is high, as it is not stated who assessed radiographic information and if they were blinded to all the indexes. Applicability concerns are low.

Flow and timing: The risk of bias is low, because CXR was made after auscultation.

Badgett et al.[29]

Selection: The risk of selection bias is high, because patients were recruited with a self-reported diagnosis of chronic bronchitis, emphysema or asthma and exclusions are unclear. Patients gave written informed consent, selecting only patients willing to contribute to clinical studies. Applicability concerns are low, because included patients match the review question.

Index test: The risk of bias is low, because the four physicians performing auscultation were blinded to patient medical history spirometric results. Applicability concerns are low.

Reference test: The risk of bias is unclear, because authors did not mention who performed sperometric testing. Applicability concerns are low.

Flow and timing: The risk of bias is unclear, because it is unclear if spirometry was performed before or after physical examination.

Badgett et al.[28]

Selection: The risk of selection bias is high, because patients were recruited with a self-reported diagnosis of chronic bronchitis, emphysema or asthma and exclusions were unclear. Patients gave written informed consent, selecting only patients willing to contribute to clinical studies. Applicability concerns are low, because included patients match the review question.

Index test: The risk of bias is low, because the four physicians performing auscultation were blinded to patient medical history spirometric results. Applicability concerns are low.

Reference test: The risk of bias is unclear, authors did not mention who performed sperometric testing. Applicability concerns were low.

Flow and timing: The risk of bias is unclear, because it is unclear if spirometry was performed before or after physical examination.

Garcia-Pachon et al.[34]

Selection: The risk of selection bias is high, because only patients older than 40 years and with more than 20 pack-years of smoking were included. Moreover, patients were already diagnosed or had a self-reported diagnosis of COPD. However, patient recruitment was consecutive and exclusions were reasonable. Applicability concerns are unclear, because a selection of included patients was made (older than 40 years and more than 20 packyears of smoking).

Index test: The risk of bias is low, because the physicians performing auscultation were blinded to spirometric results and independently documented their findings without communication. Also the performance of a first-year resident in family medicine and a experienced pulmonologist were compared. The exact number of performing physicians is unknown. Applicability concerns are low.

Reference test: The risk of bias is low, because spirometry was performed in a separate room by a trained technician blinded to any other data. Applicability concerns are low.

Flow and timing: The risk of bias is low, because spirometry was performed immediately after auscultation.

Holleman et al.[30]

Selection: The risk of selection bias is low, because patient recruitment was consecutive and exclusions were reasonable. Applicability concerns are high, because included patients were only male.

Index test: The risk of bias is low, the physicians who performed auscultation were blinded to spirometric results of the subjects. Applicability concerns are low.

Reference test: The risk of bias is low, because spirometry was performed by a physician blinded for clinical information and the results of physical examination. Applicability concerns are low.

Flow and timing: The risk of bias is low, because spirometry was performed after physical examination.

King et al.[35]

Selection: The risk of selection bias is high, patient recruitment was consecutive and exclusions were reasonable. However patients were referred because of clinical suspicion of asthma but with (nearly) normal spirometry. Applicability concerns are low, because included patients match the review question.

Index test: The risk of bias is low, the physicians who performed auscultation were blinded to spirometric results of the subjects. The performance of the physicians was covered by an investigator that examined all subjects. The exact number of performing physicians is unknown. Applicability concerns are low.

Reference test: The risk of bias is unclear, because it is unknown who performed the spirometry and if spirometry was performed with knowlegde of the results of auscultation. Applicability concerns are low.

Flow and timing: The risk of bias is low, because all tests were performed on the same day.

Leuppi et al.[31]

Selection: The risk of selection bias is low, because patient recruitment was consecutive and exclusions were reasonable. Applicability concerns are low, because included patients match the review question.

Index test: The risk of bias is low, because the physicians performing auscultation were blinded to spirometric results. Applicability concerns are low.

Reference test: The risk of bias is low, because authors did not mention who performed sperometric testing, but spirometric is considered an objective measurement. Applicability concerns are low.

Flow and timing: The risk of bias is low, because spirometry was performed after auscultation and within two hours after admission to the Emergency Department.

Ma et al.[36]

Selection: The risk of selection bias is high, because patient recruitment was retrospective. Exclusions were reasonable. Applicability concerns are low, because included patients match the review question.

Index test: The risk of bias is unclear, because it is unknown who performed the physical examination, if these performers were blinded to the spirometric results and if they used the same criteria for auscultation. Applicability concerns are low.

Reference test: The risk of bias is unclear, because it is unknown who performed the spirometry and if the performers were blinded to results of auscultation. Applicability concerns are low.

Flow and timing: The risk of bias is unclear, because it is unknown if spirometry was performed before or after physical examination.

Melbye et al.[38]

Selection: The risk of selection bias is low, because patient recruitment was consecutive and exclusions were reasonable. Applicability concerns are low, because included patients match the review question.

Index test: The risk of bias is low, because the physicians performing auscultation were blinded to spirometric results. The exact number of permorming physicians is unknown. Applicability concerns are low.

Reference test: The risk of bias is low, because spirometry was performed by specially trained nurses. Applicability concerns are low.

Flow and timing: The risk of bias is high, because time between reference standard and index test is not mentioned. Moreover, only 398 of the 574 included patients received index test, it is not stated why remaining 176 were not auscultated.

Pratter et al.[37]

Selection: The risk of selection bias is high, because it is unknown if patient recruitment was consecutive. Moreover, symptoms of included patients had been difficult to control for an average of 3.3 years. Exclusions were reasonable. Applicability concerns are low, because included patients match the review question.

Index test: The risk of bias is low, because the investigators performing auscultation were blinded to spirometric results. Applicability concerns are low.

Reference test: The risk of bias is unclear, because it is unknown who performed the spirometry and if the performers were blinded to results of auscultation. Applicability concerns are low.

Flow and timing: The risk of bias is unclear. Spirometry was performed after auscultation. However the exact timeframe is unknown.

Oshaug et al.[32]

Selection: The risk of selection bias is high, because patients were recruited by a mailed invitation. Only patients were included who aswered and consented. Applicability concerns are low, because included patients match the review question.

Index test: The risk of bias is unclear, because it is unclear if the GP's performing auscultation were blinded to spirometric results. Applicability concerns are low.

Reference test: The risk of bias is low, because spirometry was performed by trained health-care workers blinded for clinical information and the results of physical examination. Applicability concerns are low.

Flow and timing: The risk of bias is unclear, because spirometry was performed after physical examination in four GP offices, in the other three spirometry was performed before physical examination.

Straus et al.[33]

Selection: The risk of selection bias is high, because patient recruitment is unclear among the various centers. Applicability concerns are low, because included patients match the review question.

Index test: The risk of bias is low, because the two investigators performing auscultation were blinded to spirometric results. Applicability concerns are low.

Reference test: The risk of bias is low, because spirometry was performed by a physician blinded for clinical information and the results of physical examination. Applicability concerns are low.

Flow and timing: The risk of bias is low, because spirometry was performed in a good time frame within 30 minutes after physical examination.

Tomita et al.[39]

Selection: The risk of selection bias is unclear, because it is unknown if patient recruitment was consecutive. Exclusions were reasonable. Applicability concerns are low, because included patients match the review question.

Index test: The risk of bias is low, because the physicians performing auscultation were blinded to spirometric results. The exact number of permorming physicians is unknown. Applicability concerns are low.

Reference test: The risk of bias is high, because pulmonologist made the diagnosis based on spirometric and physical examination, including auscultation. Applicability concerns are low.

Flow and timing: The risk of bias is low. Spirometry was performed after auscultation at visit one.

Diehr et al.[40]

Selection: The risk of selection bias is low, because patient recruitment was consecutive. Applicability concerns are low, because included patients match the review question, although more severely ill patients were excluded.

Index test: The risk of bias is unclear, because the number of performing physicians was unclear. The investigators performing auscultation were blinded to CXR results. Applicability concerns are low.

Reference test: The radiologists who analysed the CXR was blinded to auscultatory results. The risk of bias is high, because CXR is not the gold standard for detecting pneumonia, but it is used as first line diagnostic tool in these patients. Applicability concerns are low.

Flow and timing: The risk of bias is unclear, because it is unclear when auscultation was performed immediately after or before making the CXR.

Ebrahimzadeh et al.[41]

Selection: The risk of selection bias is high, because it concerns a case-control study design and patients were chosen by physicians judgement. Applicability concerns are low, because included patients match the review question.

Index test: The infectious disease specialist performing physical examination was blinded for CXR results. The risk of bias is high, because this investigator did all the 840 examinations alone. Applicability concerns are low.

Reference test: A boardcertified radiologist analysed the CXR without knowledge of any clinical information. No thoracic CT was made, so risk of bias is high. Applicability concerns are low.

Flow and timing: The risk of bias is low, because auscultation was performed before making the CXR

Gennis et al.[42]

Selection: The risk of selection bias is high, because patients were included when there was a clinical need for CXR. Moreover, patients were excluded when a lacking data for clinical signs. Applicability concerns are low.

Index test: The risk of bias is high, because physical signs were recorded as absent when not assessed. The number of performing physicians was unclear. Applicability concerns are low.

Reference test: Authors did not mention if auscultatory results and clinical data were available for the independent radiologist. Moreover, CRX showing positive or equivoval outcome were considered to have pneumonia. Small pulmonary abnormalities could have been missed because no thoracic CT has been made, so risk of bias is high. Applicability concerns are low.

Flow and timing: The risk of bias is unclear, because it is unclear when auscultation was performed immediately after or before making the CXR.

Flanders et al.[43]

Selection: The risk of bias is low, because consecutive adults seeking care at the emergency department or acute care ambulatory clinic with acute cough were included. Applicability concerns are low, because included patients match the review question.

Index test: The risk of bias is low, because pulmonary auscultation was performed before the CXR. Applicability concerns are low.

Reference test: The risk of bias is unclear, because it is not stated whether the staff radiologist was unaware of the physical examination findings. Applicability concerns are low.

Flow and timing: The risk of bias is unclear, because the time between pulmonary auscultation and the CXR is not stated. Also, CXR had not been performed on all patients without pneumonia, but these patients were excluded if they had been treated with antibiotics, or had a diagnosis of pneumonia at a follow-up visit.

Heckerling et al.[44]

Selection: The risk of bias is low, because patient recruitment was consecutive and exclusion criteria were reasonable. Applicability concerns are low, because included patients match the review question.

Index test: The risk of bias is low, because pulmonary auscultation was performed before the CXR and recorded before obtaining the results of the CXR. Applicability concerns are low.

Reference test: The risk of bias is unclear, because it is not stated whether the attending or resident radiologist interpreted the results without knowledge of the results of auscultation. Applicability concerns are low.

Flow and timing: The risk of bias is unclear, because it was unclear when the CXR were taken.

Hopstaken et al.[45]

Selection: The risk of bias is low, because patient recruitment was consecutive and exclusion criteria were reasonable. Applicability concerns are low, because included patients match the review question.

Index test: The risk of bias is low, because pulmonary auscultation was performed before the CXR. Applicability concerns are low.

Reference test: The risk of bias is low, because the radiologists were blinded to the clinical status of the patient. Moreover, the results were re-assessed by a second and third radiologist. Applicability concerns are low. Applicability concerns are low.

Flow and timing: The risk of bias is low, because CXR were made on the third day after inclusion to ensure that possible infiltrates were detectable on the CXR.

Melbye et al.[46]

Selection: The risk of selection bias is low, because patient recruitment was consecutive and exclusion criteria were reasonable. Applicability concerns are low, because included patients match the review question.

Index test: The risk of bias is low, because pulmonary auscultation was performed before the CXR. Applicability concerns are low.

Reference test: The risk of bias is unclear, because it was unclear if the two independent radiologists were blinded to the index tests. Applicability concerns are low.

Flow and timing: The risk of bias is high, because not all patients received a CXR.

Minnaard et al.[47]

Selection: The risk of bias is low, because consecutive adults who met the broad inclusion criteria were included. Applicability concerns are low, because included patients match the review question.

Index test: The risk of bias is unclear, because it was unclear if the investigators performing auscultation were blinded to CXR results. Applicability concerns are low.

Reference test: The risk of bias is low, because the radiologists were blinded to the patients' clinical information. Applicability concerns are low.

Flow and timing: The risk of bias is unclear, because CXR were taken in a wide range of seven days of the initial presentation.

Nakanishi et al.[48]

Selection: The risk of bias is low, because consecutive patients with symptoms of new lower respiratory tract infections were included. Applicability concerns are low, because included patients match the review question.

Index test: The risk of bias is low, because pulmonary auscultation was performed before the CXR. Applicability concerns are low.

Reference test: The risk of bias is low, because the two expert respiratory physicians were blinded to the clinical information and diagnosis. Applicability concerns are low.

Flow and timing: The risk of bias is high, because the interval between the index test and the reference standard is unclear and not all patients received the same reference standard.

Reissig et al.[49]

Selection: The risk of bias is high, because patients with hospital-acquired pneumonia were excluded. Applicability concerns are low, because included patients match the review question.

Index test: The risk of bias is low, because pulmonary auscultation was performed before the CXR or CT scan was made. Applicability concerns are low.

Reference test: The risk of bias is unclear, because it is not clear whether the reference standard results were interpreted without knowledge of the results of pulmonary auscultation. Applicability concerns are low.

Flow and timing: The risk of bias is unclear, because all patients underwent CXR. However, in the case of inconclusive radiographic or positive sonography findings and of negative radiographic findings, a low-dose CT scan was performed. If other diagnosis were suspected, a spiral/multislice CT scan with contrast agent was performed.

Song et al.[50]

Selection: The risk of bias is high, because it concerns a case-control study. Applicability concerns are low, because included patients match the review question.

Index test: Risk of bias is high, because it is unclear if the index test results were interpreted without knowledge of the results of the reference standard and it is not stated who performed auscultation. Applicability concerns are low.

Reference test: The risk of bias is unclear, because it is not clear whether the reference standard results were interpreted without knowledge of the results of pulmonary auscultation. Applicability concerns are low.

Flow and timing: The risk of bias is unclear, because the interval between pulmonary auscultation and CXR or CT-scan is not mentioned. Also, CT scans were taken in all subjects with unremarkable CXR findings to exclude pneumonia development.
